# Identifying and developing strategies for implementation of a guided internet- and mobile-based infant sleep intervention in well-baby and community mental health clinics using group concept mapping

**DOI:** 10.1186/s12913-024-10632-w

**Published:** 2024-02-07

**Authors:** Filip Drozd, Hege Pettersen Sandtrø, Turid Skjerve Leksbø, Silje Marie Haga, Heidi Jacobsen, Hege Therese Størksen

**Affiliations:** 1 Regional Centre for Child and Adolescent Mental Health, Eastern and Southern Norway, N-0405 Nydalen, Oslo, PO Box 4623, Norway; 2 VID Specialized University, Oslo, Norway

**Keywords:** Implementation strategy, Nursing, Parenting, Primary care, Treatment

## Abstract

**Background:**

This study aimed to identify strategies for the implementation of a guided internet- and mobile-based intervention (IMI) for infant sleep problems (“*Sleep Well, Little Sweetheart*”) in well-baby and community mental health clinics.

**Study design:**

We used group concept mapping, a two-phased mixed methods approach, conducted as a two-day workshop in each clinic. We recruited 20 participants from four clinics and collected sorting and rating data for implementation strategies based on the Expert Recommendations for Implementing Change taxonomy and brainstorming sessions. Data were analyzed using descriptive statistics, multidimensional scaling, and hierarchical cluster analysis to create cluster maps, laddergrams, and Go-Zone graphs. Participants were presented with the results and discussed and interpreted the findings at each of the clinics in spring 2022.

**Results:**

Participants identified 10 clusters of strategies, of which *Training*, *Embedding and Coherence*, *User Involvement and Participation*, and *Clinician Support and Implementation Counseling* were rated as most important and feasible. *Economy and Funding* and *Interactive and Interdisciplinary Collaboration* were rated significantly lower on importance and feasibility compared to many of the clusters (all *p*s < 0.05). There was a correlation between the importance and feasibility ratings (*r* =.62, *p* =.004).

**Conclusions:**

The use of group concept mapping made it possible to efficiently examine well-baby and community clinics’ perspectives on complex issues, and to acquire specific knowledge to allow for the planning and prioritization of strategies for implementation. These results suggest areas of priority for the implementation of IMIs related to infant sleep problems.

**Trial registration:**

The study was pre-registered at Open Science Framework (www.osf.io/emct8).

**Supplementary Information:**

The online version contains supplementary material available at 10.1186/s12913-024-10632-w.

## Background

About 20% of infants and toddlers experience sleep problems related to sleep onset, night waking, and sleep scheduling (i.e., pediatric insomnia) [1, 2]. Few parents, however, raise their concerns about their child’s sleep problems with health professionals (HPs) and many HPs have little or no formal training in pediatric sleep [1]. There are effective psychosocial and behavioral interventions [3, 4], but their descriptions in research often lack in detail to help HPs in their implementation [5]. This may result in underidentification and undertreatment of pediatric sleep problems [1], and the endorsement and delivery of sleep assessment and treatments that are not evidence-based [6]. Thus, HPs and parents would benefit from easy access to standardized and evidence-based screening and intervention programs.

Digital interventions can provide standardized, evidence-based and accessible care ‘where parents are’, that is, in their local communities, at home, and online. Parents actively search for information about infant sleep and health online [7, 8], and most parents and HPs are interested in internet-based infant sleep programs [8, 9]. Despite this, research on internet- and mobile-based interventions (IMIs) for pediatric insomnia in typically developed young children is still in its infancy. There is currently only one randomized trial of an internet intervention, that showed reductions in problematic sleep, sleep onset latency, and the number and duration of night wakings [10]; improvements that were maintained one year later [11]. In terms of mobile interventions, only one app was empirically supported [12], according to a recent review [13]. Thus, the few initial results seem promising. A few studies have also examined the dissemination of online sleep information and the usability of online tools or interventions [14–17]. Such studies are useful for identifying barriers and facilitators to implementation such as the parental needs for professional guidance and cultural adaptations (e.g., many practice co-sleeping) and the time restrictions and training needs of HPs [15].

Internet interventions have existed for 20 years. However, with a few exceptions [18], efforts to integrate these into routine practice have had mixed success. There are many factors that may promote or impede the implementation of IMIs. Provision of information, training, and infrastructure to those involved is key to success [19], but is insufficient by itself. Many HPs may still be skeptical about using IMIs, and experience excessive workloads and disruptions to their workflow [19, 20]. Compatibility (or lack thereof) with existing systems, ambiguous policies, and costs, are other known challenges that must be addressed at an organizational or policy level [19–21]. Practical guidelines may provide important insights and key points to consider (e.g., privacy, funding schemes, policy, and regulatory context) [22, 23]; however, the development of IMIs still requires careful consideration of the context in which they are to be used [24].

To integrate IMIs into practice, it is necessary to identify and select contextually appropriate implementation strategies. Implementation strategies can be defined as “*methods or techniques used to enhance the adoption, implementation, and sustainability of a clinical program or practice*” [25, p. 2]. However, selecting implementation strategies is challenging for several reasons; it requires careful consideration of contextual variations [24, 26]; despite there being around 170 implementation theories and models [27], most have a limited evidence-base due to being underutilized [28]; and, there is an abundance of strategies that can be combined in numerous ways [29–31]. The literature thus provides limited guidance on the selection of implementation strategies. Therefore, the objective of this study was to advance our practical understanding of barriers and facilitators that can influence the implementation of IMIs. More specifically, our aim was to identify implementation strategies that are important and feasible to integrate a guided IMI for infant sleep problems (“*Sleep Well, Little Sweetheart*”) into well-baby and community mental health clinics by using group concept mapping [32, 33].

## Methods

Group concept mapping (GCM) is a two-phased, participatory sequential mixed methods approach to guide planning and program evaluation [34]. In the first qualitative phase (i.e., the first workshop), participants received a demonstration of *Sleep Well, Little Sweetheart* and explained that “*the goal of the workshop is to arrive at a framework for the implementation of the program*”. They were specifically instructed to “*consider the strategies related to Sleep Well, Little Sweetheart*”, although the focus prompt was formulated as “*what conditions must be present for successful implementation at your workplace?*”, to help with the sorting and rating tasks, and to broaden their mind during brainstorming. Finally, participants individually sorted and rated a set of pre-defined implementation strategies and engaged in a brainstorming session (see Supplementary Materials). In the second phase, researchers performed quantitative analyzes to represent the sorting and rating data, which were presented at the second workshop where participants discussed and interpreted the findings.

### Intervention

*Sleep Well, Little Sweetheart* is a guided IMI for pediatric insomnia in infants from six months to three years. It consists of eight program modules: (1) infant sleep assessment [35], (2) psychoeducation about infant sleep [35, 36], (3) individual bedtime routine [37], (4) infant crying [38], (5) parent emotion regulation [39], (6) individual sleep plan (i.e., extinction-based plans, bedtime fading or scheduled awakenings) [40], (7) relapse prevention, and (8) a sleep diary [41]. The program is administered and delivered by HPs, using the Youwell platform (www.youwell.no). HPs task is to establish and maintain a high-quality working alliance with the parents, motivate them to use the program, and adapt the program contents to the individual family (e.g., individual bedtime routine and sleep plan) [42], either during routine face-to-face consultations or via technology (i.e., text messages or mobile phone).

### Participants

We recruited 20 participants from two well-baby clinics (*n* = 13, 65%) and two community mental health clinics (*n* = 7, 35%), which is sufficient for GCM and above the typically recommended sample size of 15 [43]. All participants were women, with an average age of 47.1 years, and a university or college degree. Eleven (55%) of the participants worked as public health nurses (PHNs), four (20%) as psychologists, and four (20%) in other positions (e.g., family therapists). Three (15%) of the participants were clinic leaders. The majority had some clinical training and experience with infant sleep (*n* = 18, 90%), but few had any personal or professional experience with IMIs (*n* = 4, 20%).

### Data collection

Prior to the first workshop, participants gave their consent and provided background information (e.g., age, employment, and experience with infant sleep). GCM was conducted in each clinic from March to May/June 2022. The first step of data collection consisted of individual sorting of 73 implementation strategies from the Expert Recommendations for Implementing Change (ERIC)–taxonomy [29], including suggested labels for each group of piles. Participants were instructed to group strategies in a meaningful way, based on their similarities. The participants then rated each strategy in terms of its relative importance and feasibility in their workplace, as two separate ratings, on a scale from 1 (“*not at all important/feasible*”) to 5 (“*extremely important/feasible*”). To ensure that the piles were not mixed, participants were instructed to use rubber bands and zipped bags (see Supplementary Materials for participant instructions).

The ERIC–taxonomy is a widely applicable, standardized, and manageable set of implementation strategies that minimizes participant burden and maximizes breadth. However, it may not cover context-specific strategies outside U.S. or North–American settings and ensure data saturation. Thus, participants brainstormed individually and created 56 additional strategies that were written on post-its and placed in separate zipped bags. These were reduced to 24 novel strategies by plenary discussions and a final review by the second author, which can be considered indicative of saturation. The list of statements and translations is provided in Table 1. At the second workshop, participants were presented with the results from the first workshop, they discussed and interpreted the findings (e.g., if the results were surprising or reflected their opinion), and sorted and rated strategies from the brainstorming. Analyses were then updated to include sorting and rating data from the second workshop. The participants’ interpretations of the findings were audio-recorded, summarized, and integrated in the Discussion below. The first and second authors moderated the workshops.

**Table 1:** Overview and translations of implementation strategies.^a^

#	Implementation Strategies (Norwegian)	Definitions (Norwegian)	Implementation Strategies (English)	Definition of Additional Strategies (English)
1	Sikre finansiering	Sikre tilgang til nye eller eksisterende midler for å tilrettelegge for implementeringen (f.eks. tilskudd, øremerking og nye betalingstjenester)	Access new funding	
2	Endre insentiv- og godtgjørelsesordninger	Sikre ordninger for å stimulere til bruk og implementering av [tiltaket] (f.eks. lønnspålegg, forfremmelse og nye ansvarsområder)	Alter incentive/allowance structures	
3	Endre betalings- og/eller avgiftsordninger	Lag ordninger der brukere betaler mindre for bruk av [tiltaket] enn for andre behandlingsalternativer	Alter patient/consumer fees	
4	Kartlegg mottakelighet og potensielle hemmere og fremmere	Kartlegg hvor mottakelig tjenesten er for praksisendringer, identifiser barrier/hindringer og drivere/fasilitatorer for implementering (f.eks. arbeidsklima, ressurser og lederskap)	Assess for readiness and identify barriers and facilitators	
5	Utfør en kritisk gjennomgang (revisjon) og gi tilbakemeldinger	Samle inn og oppsummere kliniske data og gi dem til praktikere og administrasjonen for å holde øye med, evaluere og endre måten ansatte jobber på	Audit and provide feedback	
6	Opprett gjensidige samarbeidsforhold eller koalisjoner	Etabler og oppretthold relasjoner med partnere i implementeringsarbeidet (f.eks. beslutningstakere, samarbeidspartnere, undervisere og brukerorganisasjoner)	Build a coalition	
7	Fang opp og del lokal kunnskap	Fang opp erfaringer og kunnskap fra tjenester som har lykkes/ikke lykkes med implementeringen og lær av dem. Del erfaringene mellom tjenester	Capture and share local knowledge	
8	Sentraliser teknisk support	Utvikle og bruk et sentralisert system for teknisk hjelp og støtte knyttet til implementeringen av [tiltaket] (f.eks. epost, nettside og hjelpetelefon)	Centralize technical assistance	
9	Still krav til akkreditering eller medlemskap	Få på plass krav og standarder slik at de som ønsker å tilslutte seg [tiltaket] blir oppmuntret eller pålagt å bruke den (f.eks. Ammekyndig helsestasjon)	Change accreditation or membership requirements	
10	Endre lovverk, fagprosedyrer, retningslinjer og veiledere	Delta i arbeid med å endre ulike kilder til informasjon som gjør praktikere mer villige til å tilby [tiltaket]	Change liability laws	
11	Endre fysiske omgivelser og utstyr	Evaluer og tilpass, etter behov, de fysiske innretningene og/eller utstyret for best mulig å imøtekomme [tiltaket] (f.eks. endre utformingen av et rom, innkjøp av utstyr)	Change physical structure and equipment	
12	Endre journalsystemer	Gjør endringer i journalsystemer for bedre vurdering av implementeringen eller klientarbeidet som følge av praksisendringene	Change record systems	
13	Endre tjenestestedets lokasjon	Flytt tjenesten for økt tilgang på kunnskap og kompetanse, tverrfaglig/-etatlig samarbeid, eller bringe tjenestetilbudet ut til hjemmene, samfunnet eller andre relevante omgivelser for brukerne	Change service sites	
14	Gjennomfør små og jevnlige pilottester	Implementer små endringer av gangen og gjennomfør jevnlige tester for innsikt i hvordan gjøre det bedre, før praksisendringene settes ut i hele tjenesten	Conduct cyclical small tests of change	
15	Arranger opplæringsmøter	Arranger møter med forskjellige interessenter for opplæring i [tiltaket] (f.eks. ansatte, ledere, familier, brukerorganisasjoner, frivillige)	Conduct educational meetings	
16	Få oppsøkende opplæringsbesøk	Få en [tiltaket]-trener til å møte tjenesten (fysisk, digitalt og hybrid) i deres praksismiljø for å utdanne ansatte i [tiltaket] med den hensikt å endre tjenestens praksis	Conduct educational outreach visits	
17	Gjennomfør lokale konsensus-diskusjoner	Inkluder ansatte i avgjørelser som tar for seg viktigheten av problemet og om [tiltaket] er hensiktsmessig for å løse problemet	Conduct local consensus discussions	
18	Gjennomfør en lokal behovsvurdering	Samle inn opplysninger og analyser behovet for [tiltaket] (f.eks. omfang av problem, (mangelfull) kompetanse hos ansatte, tjenestetilbud)	Conduct local needs assessment	
19	Gi opplæring over tid	Planlegg for og gjennomfør opplæringen i [tiltaket] jevnlig over tid (inkl. veiledning, ferdighetstrening, boostere, for nyansatte og viderekomne, osv.)	Conduct ongoing training	
20	Opprett praksis-/læringsnettverk	Tilrettelegg for ansattgrupper innad i, eller på tvers av, tjenester for å fremme et samarbeidende læringsmiljø og styrke implementeringen	Create a learning collaborative	
21	Opprett nye praksisteam/kliniske faggrupper	Endre hvem som jobber i teamet, legg til ulike yrkes-/faggrupper og ferdigheter for å gjøre det mer sannsynlig at [tiltaket] blir brukt på en vellykket måte	Create new clinical teams	
22	Opprett kvalifikasjons- og/eller sertifiseringskrav	Lag sertifiserings- eller lisenskrav for bruk av [tiltaket], krav til opplæring, vedlikeholdsaktivitet, og lignende	Create or change credentialing and/or licensure standards	
23	Lag og bruk en formell implementeringsplan	Beskriv detaljer i implementeringen om strategier, verktøy, roller og ansvarsfordeling, tidsrammer, milepæler og fremdrift. Planen bør brukes aktivt	Develop a formal implementation blueprint	
24	Opprett partnerskap med forskere og akademikere	Samarbeid med et universitet, høgskole, eller kompetansesenter, for opplæring og å få forskningsferdigheter inn i implementeringen	Develop academic partnerships	
25	Sørg for felles språkforståelse	Sikre at alle involverte i implementeringen har en felles forståelse for begreper som brukes (f.eks. lag en ordliste i fellesskap)	Develop an implementation glossary	
26	Lag verktøy for kvalitetsforbedring på individnivå	Mål kvalitet på arbeid med brukere og praksisendringer, og gi den enkelte ansatte jevnlige tilbakemeldinger på eget arbeid (f.eks. antall saker, etterlevelse og resultater i arbeid med brukere)	Develop and implement tools for quality monitoring	
27	Lag systemer for kvalitetsforbedring på systemnivå	Mål kvalitetsindikatorer på praksisendringer som følge av [tiltaket] i et register som gir oppdaterte tilbakemeldinger på implementeringsprosessen i tjenesten (f.eks. antall saker, brukerresultater)	Develop and organize quality monitoring systems	
28	Opprett sanksjonsordninger	Gi økonomiske sanksjoner for manglende implementering eller bruk av [tiltaket] (f.eks. frafall av bonus, lønnspålegg og forfremmelse)	Develop disincentives	
29	Lag opplæringsmateriell	Utvikle manualer, verktøy og annet støttemateriell som gjør det lettere å lære om [tiltaket], og lettere for praktikere å levere [tiltaket] til brukere	Develop educational materials	
30	Lag avtaler om ressursdeling	Inngå partnerskap med andre som har ressurser som trengs for å implementere [tiltaket] (f.eks. kommuner, IT, utstyrsleverandører, frivillige)	Develop resource sharing agreements	
31	Distribuer opplæringsmateriell	Distribuer opplæringsmaterialet (inkl. veiledere, manualer, verktøy, osv.) personlig, per post, og/eller elektronisk	Distribute educational materials	
32	Tilrettelegg for formidling av kliniske data til praktikere	Overfør eller gi ansatte tilgang til opplysninger samlet inn fra brukeren der data vanligvis ikke samles inn i konsultasjon (f.eks. aktivitet, søvn og måltider)	Facilitate relay of clinical data to providers	
33	Tilrettelegg for [tiltaket]	Anerkjenn behovet for [tiltaket], et støttende implementeringsklima og for å løse problemer som oppstår i fellesskap	Facilitation	
34	Sørg for stabil finansiering og politisk støtte	Jobb for at myndigheter stiller seg bak [tiltaket], oppfordrer til å ta den i bruk og utvikler nye finansieringsordninger	Fund and contract for the clinical innovation	
35	Identifiser og forbered superbrukere	Identifiser og forbered ansatte som dedikerer seg til å markedsføre, støtte, være pådrivere og overvinne likegyldighet eller motstand i implementeringen	Identify and prepare champions	
36	Identifiser tidlige brukere («early adopters»)	Se etter særlig engasjerte ansatte/tjenester som hurtig tar til seg [tiltaket], og som kan bistå i implementeringen, og ta lærdom av erfaringene deres	Identify early adopters	
37	Øk etterspørselen	Markedsfør eller gi informasjon og opplæring om [tiltaket] rettet mot relevante målgrupper (f.eks. lokalsamfunn, familier og samarbeidspartnere)	Increase demand	
38	Informer lokale nøkkelpersoner om [tiltaket]	Informer personer som blir ansett av kollegaer som betydningsfulle og innflytelsesrike, om [tiltaket] i håp om at de vil påvirke kollegaer til å ta den i bruk	Inform local opinion leaders	
39	Sørg for brukermedvirkning for økt bruk av [tiltaket]	Utvikle strategier med brukere for å oppmuntre til og løse problemer knyttet til bruk av [tiltaket]	Intervene with patients/consumers to enhance uptake and adherence	
40	Involver styrings- og ledergrupper	Engasjer eksisterende styringsstrukturer i implementeringsarbeidet (f.eks. politikere, administrativ ledelse, fagledere og andre beslutningstakere)	Involve executive boards	
41	Involver brukere og familier i implementeringen	Engasjer brukere og familier for tilbakemeldinger og evaluering av implementeringsarbeidet	Involve patients/consumers and family members	
42	Gjør fakturering enklere	Gjør det enklere å fakturere for [tiltaket] (f.eks. forenklede dokumentasjonskrav)	Make billing easier	
43	Gjør opplæringen dynamisk og interaktiv	Varier undervisningen for å imøtekomme ulike læringsstiler og arbeidsforhold, og sikre at deltakere bidrar aktivt til opplæringen	Make training dynamic	
44	Sikre ledelsens mandat for praksisendringene	Sørg for at ledelsen prioriterer [tiltaket], viser endringsvilje og gir sin tilslutning og sitt mandat til gjennomføringen av [tiltaket] og de planlagte praksisendringene	Mandate change	
45	Modeller og simuler endring	Demonstrer, vis eller på andre måter gjennomgå endringene som skal iverksettes, før implementering	Model and simulate change	
46	Innhent og bruk tilbakemeldinger fra brukere og deres familier	Utvikle strategier for å innhente tilbakemeldinger fra brukere (f.eks. brukertilfredshet, klager, avvik og behandlingsresultater)	Obtain and use patients/consumers and family feedback	
47	Få formelle forpliktelser	Lag skriftlige avtaler med nøkkelpersoner/-organisasjoner som beskriver hva de vil gjøre for å implementere [tiltaket]	Obtain formal commitments	
48	Arranger implementeringsmøter med ansatte	Organiser ansatte som implementerer [tiltaket]. Gi de tid og mulighet til å reflektere rundt implementeringen, dele erfaringer og støtte hverandres læring	Organize clinician implementation team meetings	
49	Sørg for refusjonsordninger for [tiltaket]	Arbeide for at tjenestene, praktikere eller brukere kan få refusjon for [tiltaket]	Place innovation on fee for service lists/formularies	
50	Forbered brukere på å være aktive deltakere	Forbered brukere på å være aktive, stille spørsmål, etterlyse informasjon om [tiltaket], samt inviteres inn til beslutninger vedrørende egen behandling	Prepare patients/consumers to be active participants	
51	Tilpass [tiltaket] til praksis	Identifiser hvilke elementer ved [tiltaket] som kan tilpasses lokale forhold, og hvilke som er viktige for kvalitetssikring og troskap til [tiltaket]	Promote adaptability	
52	Fremme nettverksbygging	Bygg på eksisterende arbeidsrelasjoner av høy kvalitet i og utenfor organisasjonen for å fremme informasjonsdeling, problemløsning og en felles visjon/mål knyttet til implementering av [tiltaket]	Promote network weaving	
53	Tilby praktisk/klinisk veiledning	Gi opplæring til veiledere som skal gi løpende veiledning til praktikere, og tilby veiledning i [tiltaket]	Provide clinical supervision	
54	Tilby lokal hjelp og støtte	Lag et system med lokalt ansatte (dvs. koordinator) som kan assistere i implementeringsprosessen (1. linjesupport), og evt. be om hjelp utenfra (2. linjesupport)	Provide local technical assistance	
55	Tilby fortløpende konsultasjoner	Konsulter med en eller flere eksperter i strategiene som brukes for å støtte implementering av [tiltaket]	Provide ongoing consultation	
56	Evaluer og re-vurder implementeringen	Monitorer fremdrift og juster praksis og implementeringen av [tiltaket] jevnlig	Purposely reexamine the implementation	
57	Gi opplæring i implementeringsledelse	Rekrutter, ansvarliggjør og lær opp ledere for praksisendringene som følge av [tiltaket]	Recruit, designate, and train for leadership	
58	Påminnelser for utøvere av [tiltaket]	Utvikle systemer for å hjelpe praktikere med å huske informasjon og/eller minne om bruk av [tiltaket] (f.eks. varsler om oppfølging av brukere eller brukermeldinger)	Remind clinicians	
59	Revider stillingsbeskrivelser	Re-designe og endre roller, oppgaver og ansvarsområder blant praktikere som jobber med [tiltaket]	Revise professional roles	
60	Lær ved å skygge andre eksperter	La nøkkelpersoner observere erfarne praktikere jobbe med praksisendringene eller bruke [tiltaket]	Shadow other experts	
61	Skaler opp implementeringen trinnvis	Fase inn implementeringen med små piloter eller utprøvinger og gradvis gå over til en mer systemomfattende utrulling	Stage implementation scale up	
62	Start en egen forening for [tiltaket]	Finn eller start en interesseorganisasjon som kan være faglig ansvarlig for opplæring, veiledning, sertifisering, oppdateringer, videreutvikling, mv. av [tiltaket]	Start a dissemination organization	
63	Skreddersy implementeringsstrategier	Tilpass strategier for å håndtere kartlagte utfordringer og muligheter basert på innsamlede opplysninger, og erfaringer underveis	Tailor strategies	
64	Bruk referanse-, rådgivnings- og/eller arbeidsgrupper	Organiser og engasjer forskjellige formelle grupper av interessenter som kan gi råd og innspill til implementeringsprosessen og foreslå forbedringer	Use advisory boards and workgroups	
65	Bruk implementeringsrådgiver	Søk rådgiving fra eksperter på implementering	Use an implementation advisor	
66	Gi økonomisk kompensasjon	Reduser eller endre kostnadene og/eller lisensutgifter knyttet til bruk av [tiltaket] (f.eks. etter en viss måloppnåelse)	Use capitated payments	
67	Bruk ekspertise for håndtering av dataopplysninger	Involver, ansett eller konsulter eksperter for å veilede ledelsen i bruk av opplysninger fremkommet i implementeringsarbeidet	Use data experts	
68	Bruk data fra ulike kilder for støtte til beslutninger	Integrer opplysninger fra journaler, [tiltaket], og andre kilder for å lette implementeringen på tvers av systemer	Use data warehousing techniques	
69	Bruk massemedier	Bruk media for å nå ut til et stort antall mennesker, markedsføre og spre ordet om [tiltaket]	Use mass media	
70	Bruk andre insentiv-/kompensasjonsordninger	Lag ordninger som frigjør praktikeres tid, og som motiverer til bruk av [tiltaket]	Use other payment schemes	
71	Gi opplæring til trenere («train-the-trainer»)	Lær opp bestemte ansatte slik at de kan gi lokal opplæring i tjenesten i/om [tiltaket]	Use train-the-trainer strategies	
72	Besøk eller hospiter hos andre tjenester	Besøk eller hospiter hos andre tjenester der lignende implementering er ansett som vellykket	Visit other sites	
73	Samarbeid med utdanningsinstitusjoner	Oppmuntre utdanningsinstitusjoner til å undervise, lære opp og trene studenter og ansatte i [tiltaket]	Work with educational institutions	
74	Sørg for tydelig ledelsesforankring	Ansatte gis nok tid til [tiltaket], opplever implementeringen som et felles prosjekt, med et felles mål og at ledelsen fungerer som motivator	Ensure managerial support and embedding	Staff is provided with sufficient time for [the intervention], experience the implementation as a joint project with a common goal, and the management acts as a motivator
75	Gi felles opplæring for hele tjenesten	Gi obligatorisk opplæring til alle ansatte om søvn og [tiltaket] for å skape en eierskapsfølelse til programmet, og for at alle skal kunne følge opp sine egne familier ift. søvnveiledning.	Provide training for the entire clinic/service (i.e., a generalist model)	Provide mandatory training to all clinical staff on sleep and [the intervention] to create a sense of ownership of the program, enabling staff to provide their families with sleep guidance.
76	Lær ansatte å jobbe kunnskapsbasert	Gi ansatte kunnskap om hvordan de kan jobbe kunnskapsbasert med søvn i møte med familiene, og hva det innebærer for tjenesten	Teach practitioners to work according to evidence-based principles	Provide staff with knowledge about working with evidence-based sleep practices in families, and what that entails for the healthcare service
77	Opprett faste veilednings-/teammøter	Gjennomfør faste veilednings-/teammøter rundt [tiltaket] hvor fordeler/ulemper med programmet diskuteres, samt drøfting av caser fra praksis	Create regular mentoring/team meetings	Conduct regular supervisory/team meetings where the advantages/disadvantages of the program are discussed, as well as discussions of cases from practice
78	Synliggjør den relative fordelen med tiltaket	Gjør [tiltaket] kjent for brukere og helsepersonell, få frem nytten, hvordan programmet skiller seg fra andre (veiledet med skreddersøm) og hvem som tilbyr programmet	Make the relative advantage of the program visible	Make [the intervention] known to users and healthcare professionals, highlight the benefits, how the program differs from other practices (e.g., guidance) and who is offering the program
79	Lag et digitalt selvstudium	Lag en opplæringspakke med et digitalt selvstudium i bruken av [tiltaket] for større fleksibilitet og mindre ressursbruk i opplæring av ansatte	Create an e-learning course for practitioners	Create a training package with a digital self-study in the use of [the intervention] for greater flexibility and less use of resources during staff training
80	Søk tilskudds-/prosjektmidler	Gjør tjenesten kjent med muligheter for å søke tilskudd-/prosjektmidler (f.eks. Helsedirektoratet/Statsforvalteren)	Apply for grants/project funding	Make the healthcare service familiar with opportunities to apply for grants/project funds (e.g., Directorate or County Governors)
81	Opprett kvalitetssikring og sertifiseringsordning	Ha en sertifiseringsordning for [tiltaket] tilsvarende Ammekyndig helsestasjon e.l.	Create a quality assurance and certification scheme	Create a certification scheme for [the intervention] corresponding to Mother-Baby Friendly Initiative standards or similar
82	Opprett ressursteam i tjenesten	Ha ressurspersoner i tjenesten som har ansvar for opplæring og oppfølging av egne ansatte i bruken av [tiltaket] i tjenesten.	Create resource teams in the service	Have in-house supervisors/superusers responsible for training and follow-up of own staff in the use of [the intervention] at the clinic.
83	Informer ansatte om implementeringsplanen	Informer om valgte implementeringsstrategier med en tidsramme, når det er bestemt at [tiltaket] skal benyttes i tjenesten	Inform employees about the implementation plan	Inform about the implementation strategies and the timeline when it is decided that [the intervention] will be taken up in the healthcare service
84	Ivareta metodetroskap	Lag en strategi for tjenestene i hvordan opprettholde engasjement og bruk av [tiltaket] for helsepersonell og familier	Ensure program fidelity	Create a strategy for maintaining commitment and use of [the intervention] among staff and families
85	Sørg for digitalt utstyr og kunnskap	Sørg for at de ansatte har tilstrekkelige digitale ferdigheter og nødvendige digitale verktøy for å kunne bruke programmet (som bærbar PC), samt får en praktisk gjennomgang av den digitale plattformen for å levere og administrere [tiltaket]	Provide digital equipment and knowledge	Ensure that staff have sufficient digital skills and the necessary digital tools to use the program (such as laptops) and receive a hands-on review of the digital platform to deliver and manage [the intervention]
86	Veiledning i arbeid med ambivalens hos foreldre	Sørg for at det i veilederen til [tiltaket] har en del som tar for seg hvordan møte ambivalens og motstand hos foreldre	Provide counseling in working with ambivalence among parents	Ensure that [the intervention] manual addresses how to work with ambivalence and resistance in parents
87	Unngå konkurrerende aktivitet	Sørg for at implementeringen av [tiltaket] prioriteres og ikke må konkurrere med andre tiltak som tjenesten ønsker å ta i bruk	Avoid competing activities	Ensure that [the intervention] is given priority and that there are no competing activities in the clinic at the time of implementation
88	Tilpasning til juridiske rammer	Sørg for at [tiltaket] er i tråd med lovverk for tjenesten, journalsystem og dokumentasjonskrav	Ensure program is in line with legal frameworks and requirements	Ensure that [the intervention] is in line with healthcare service legislation, patient records, and requirements for documentation
89	Tilpasning til øvrige arbeidsoppgaver	Sørg for at [tiltaket] er praktisk og lett å gjennomføre og passer med andre oppgaver i tjenesten	Adapt to other work tasks	Make sure that [the intervention] is practical and easy to implement and fits with other tasks in the service
90	Gjør tiltaket kultursensitivt	Sørg for at [tiltaket] er tilpasset familier med ulik etnisk bakgrunn (f.eks. språk, bilder og innhold)	Make cultural adaptations to the program	Ensure that [the intervention] is adapted to families with different ethnic backgrounds (e.g., language, images, and content)
91	Bygg kapasitet i tjenesten	Sørg for at tjenesten/ansatte har kapasitet og ressurser til å implementere [tiltaket], spesielt med tanke på oppstartfasen og at det er et trygt arbeidsmiljø for prøving-og-feiling i prosessen	Build service capacity	Ensure that the service/staff has the capacity and resources to implement [the intervention], especially with regard to the start-up phase, and that there is a safe working environment for trial-and-error in this process
92	Opprett en koordinatorfunksjon	Sørg for en intern [tiltaket]-koordinator som bistår og har faste møter internt i bydeler/kommuner med flere helsestasjoner, og eksternt med tiltakseier.	Establish a coordinator function/role	Arrange for an internal coordinator who can assist and hold regular meetings in city districts/municipalities with several healthcare services and be the main contact to intervention providers.
93	Legg kostnader på systemnivå	Sørge for at [tiltaket] har små kostnader på kommune-, etat- eller bydelsnivå, og er gratis for tjenestene og for foreldre	Place costs at the system level	Ensure that [the intervention] has low costs at the municipal or city district level and is free of charge for services and families
94	Sørg for jevnlige faglige oppdateringer	Tiltakseier holder [tiltaket] jevnlig oppdatert og at oppdateringene kommuniseres ut til tjenestene (kvalitetssikring)	Ensure regular program and content updates	The intervention providers update [the intervention] on a regular basis and convey the updates to the services (quality assurance)
95	Utarbeid en behandlingskjede	Utarbeide en klar behandlingskjede og ha retningslinjer for oppfølging og henvisning av familier hvor [tiltaket] ikke har løst søvnproblemene.	Make a plan for treatment referrals	Prepare a treatment pathway and guidelines for referrals and follow-up of families in cases where [the intervention] has not solved the sleep problem
96	Lag en utvidet veileder	Veilederen i [tiltaket] inneholder mer informasjon om tema rundt søvn som er viktige i søvnveiledning, men som programmet ikke dekker (f.eks. morgenrutiner og samarbeid mellom foreldrene)	Create an extended program and treatment manual	[The intervention] manual contains more information on topics related to sleep that are important for guidance, but which the program does not cover (e.g., morning routines and cooperation between parents)
97	Forankre [tiltaket] i retningslinjene	Vær tydelig på hensikten med [tiltaket] (balansen mellom det forebyggende og behandlende). Gjør søvn til en prioritert oppgave og introduser [tiltaket] for foreldre i en bestemt konsultasjon på helsestasjonen.	Embed the program in national/professional guidelines	Be clear about the purpose of [the intervention] (the balance between prevention and treatment). Make sleep a prioritized task and introduce [the intervention] to parents during specific consultations at the clinic

^a^ Strategies 1–73 are from the Expert Recommendations for Implementing Change (ERIC)-taxonomy (Powell, et al. 2015), while strategies 74–97 were generated during brainstorming.

### Data analyses

Demographics, importance, and feasibility ratings were analyzed using descriptive statistics. Analyses were performed using the open-source software R [44] and R-CMap package [45]. Ward’s algorithm was used for multidimensional scaling and hierarchical cluster analysis to characterize how participants grouped strategies and how they were rated in terms of their importance and feasibility (i.e., a cluster rating map). The stress value of the multidimensional scaling was 0.336 and is an indicator of the relationship between the strategies, their similarities, and distances on the map, in which lower values reflect a better fit. Values ≤ 0.39 are acceptable and unlikely to have either no structure or a random two-dimensional configuration [43, 46]. Furthermore, we calculated the split-half reliability as a measure of the overall consistency of the card sort, using 20 random splits of participants, to 0.39.

**Fig. 1:**
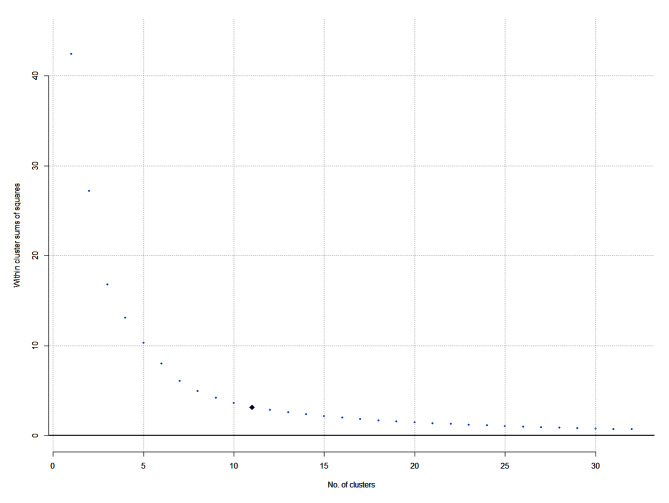
Number of clusters– within cluster sums of squares

There is no true number of clusters in a final map. The goal is to produce a set of clusters that are intuitive and meaningful. The within-cluster sum of squares, a measure of the variability of observations within each cluster, indicated an 11-cluster solution as a point of departure (see Fig. 1). A backward process with a stepwise reduction in clusters ended when further merging disrupted the meaning of the strategies in each separate cluster, as sorted by participants. It should be noted that the structure of hierarchical trees is determined by analysis, and not by the researchers [47]. In this study, the first and fifth authors examined clusters emerging from each step and agreed on the final number, after reviewing the content and meaning within each cluster. Each cluster was labeled based on the names proposed by the participants. After determining the number of clusters, each strategy’s importance and feasibility score were plotted on a scatterplot and divided into four quadrants using the mean of each dimension to identify actionable strategies for an implementation plan (i.e., ‘Go-Zone’ analyses). A laddergram was created to show cluster-level differences in importance and feasibility ratings and an analysis of variance and Tukey’s post-hoc test of multiple comparisons was used for mean comparisons between clusters.

## Results

All participants sorted and rated all strategies, except one that only sorted and rated strategies in the ERIC–taxonomy. On average, participants created 8.25 piles (*SD* = 2.92; Range = 5–16). Two participants put more than one-third of the cards in one pile. Strategies 8, 10, 11, 12, 68, and 87 formed a separate cluster in the initial 11-cluster solution but were merged to form a single cluster labeled *Preparation and Facilitation*, as the original clusters were not judged as sufficiently distinct or intuitive. The final cluster map consists of 10 clusters with 4 to 13 strategies per cluster. Table 2 presents a summary of the clusters, their corresponding strategies, and mean importance and feasibility ratings at the cluster level. Table 3 summarizes the implementation strategies and their mean importance and feasibility ratings, organized by cluster and Go-Zone quadrant.

**Table 2:** Summary of clusters of implementation strategies and their importance and feasibility ratings

			Importance		Feasibility	
Cluster	N	Strategy	Mean	Standard Deviation	Mean	Standard Deviation
1. Economy & Funding	12	1, 2, 3, 28, 34, 42, 49, 66, 67, 70, 80, 93	3.26	1.43	2.53	1.22
2. Preparation & Facilitation	13	4, 8, 10, 11, 12, 18, 23, 33, 35, 51, 68, 87, 89	3.60	1.20	3.19	1.07
3. Implementation	10	5, 14, 26, 27, 36, 56, 58, 61, 63, 92	3.46	1.01	3.08	1.01
4. Interactive & Interdisciplinary Collaboration	10	6, 7, 20, 21, 24, 52, 62, 72, 73, 81	3.21	1.12	2.97	1.08
5. Embedding & Coherence	8	9, 17, 32, 38, 45, 48, 78, 83	3.81	1.12	3.60	1.06
6. Leadership & Organization	10	13, 40, 44, 47, 59, 74, 85, 88, 91, 97	3.66	1.40	3.15	1.25
7. Training	8	15, 16, 29, 31, 43, 86, 94, 96	4.04	0.94	3.83	0.97
8. Clinician Support & Implementation Counseling	13	19, 25, 53, 54, 55, 60, 64, 65, 71, 75, 76, 77, 82	3.76	1.11	3.43	1.03
9. Quality Assurance	4	22, 57, 84, 95	3.60	1.04	3.13	1.04
10. User Involvement & Participation	9	30, 37, 39, 41, 46, 50, 69, 79, 90	3.79	1.17	3.39	1.05

**Table 3:** Summary of implementation strategies organized by Go-Zone quadrants globally and per cluster

		Importance		Feasibility			
Clusters and Statements	Strategy	Mean	Standard Deviation	Mean	Standard Deviation	Global Go-Zone	Go-Zone by Cluster
**1. Economy & Funding**							
Apply for grants/project funding	80	3.63	1.01	3.68	1.00	I	I
Access new funding	1	4.05	1.23	2.55	1.15	III	I
Fund and contract for the clinical innovation	34	4.00	1.08	2.65	1.18	III	I
Use other payment schemes	70	4.35	0.81	3.10	0.72	III	I
Place costs at the system level	93	4.53	0.61	3.00	0.67	III	I
Make billing easier	42	2.50	1.40	2.65	1.27	IV	II
Use capitated payments	66	3.25	1.37	2.55	1.32	IV	II
Use data experts	67	3.25	1.21	2.95	1.15	IV	II
Alter incentive/allowance structures	2	2.40	1.14	1.60	0.82	IV	IV
Alter patient/consumer fees	3	3.15	1.50	2.30	1.42	IV	IV
Develop disincentives	28	1.25	0.72	1.20	0.70	IV	IV
Place innovation on fee for service lists/formularies	49	2.90	1.25	2.15	1.04	IV	IV
**2. Preparation & Facilitation**							
Assess for readiness and identify barriers and facilitators	4	4.10	1.12	3.40	0.75	I	I
Develop a formal implementation blueprint	23	3.95	0.94	3.40	0.94	I	I
Facilitation	33	4.20	0.62	3.75	0.97	I	I
Identify and prepare champions	35	3.90	1.02	3.70	0.73	I	I
Avoid competing activities	87	3.63	1.01	3.42	0.90	I	I
Adapt to other work tasks	89	4.63	0.50	3.74	0.93	I	I
Conduct local needs assessment	18	3.60	1.35	3.50	1.00	II	I
Promote adaptability	51	3.35	1.23	3.35	1.04	II	II
Centralize technical assistance	8	4.05	0.94	3.20	1.06	III	I
Change liability laws	10	3.30	0.92	2.75	1.07	IV	IV
Change physical structure and equipment	11	2.15	1.39	2.40	1.19	IV	IV
Change record systems	12	3.05	1.15	2.60	1.05	IV	IV
Use data warehousing techniques	68	3.00	1.12	2.35	1.04	IV	IV
**3. Implementation**							
Identify early adopters	36	4.05	0.89	3.75	0.91	I	I
Purposely reexamine the implementation	56	3.95	0.89	3.25	0.91	I	I
Remind clinicians	58	3.80	0.62	3.70	0.92	I	I
Stage implementation scale up	61	3.30	0.98	3.30	0.92	II	II
Tailor strategies	63	3.85	0.81	3.05	0.83	III	III
Conduct cyclical small tests of change	14	2.75	1.16	3.15	1.14	IV	II
Develop and organize quality monitoring systems	27	3.50	0.76	2.90	0.79	IV	III
Audit and provide feedback	5	3.05	1.15	2.35	0.93	IV	IV
Develop and implement tools for quality monitoring	26	3.30	0.80	2.55	0.83	IV	IV
Establish a coordinator function/role	92	3.00	1.20	2.79	1.13	IV	IV
**4. Interactive & Interdisciplinary Collaboration**							
Create a learning collaborative	20	3.65	0.88	3.40	0.94	I	I
Build a coalition	6	3.75	1.12	3.15	1.14	III	I
Capture and share local knowledge	7	3.75	0.72	3.10	1.02	III	I
Develop academic partnerships	24	3.50	1.19	3.20	1.24	IV	I
Promote network weaving	52	3.45	0.89	3.20	0.83	IV	I
Create a quality assurance and certification scheme	81	3.11	1.05	3.16	0.90	IV	II
Create new clinical teams	21	2.65	1.04	2.70	1.08	IV	IV
Start a dissemination organization	62	2.35	1.23	2.40	1.23	IV	IV
Visit other sites	72	2.85	0.93	2.80	0.89	IV	IV
Work with educational institutions	73	3.00	1.30	2.65	1.18	IV	IV
**5. Embedding & Coherence**							
Conduct local consensus discussions	17	4.20	0.83	3.90	0.97	I	I
Model and simulate change	45	4.05	1.10	3.70	0.98	I	I
Organize clinician implementation team meetings	48	4.35	0.88	3.80	1.11	I	I
Make the relative advantage of the program visible	78	3.84	0.83	3.53	0.96	I	I
Inform employees about the implementation plan	83	4.00	0.82	4.00	0.88	I	I
Facilitate relay of clinical data to providers	32	3.45	1.23	3.25	1.02	II	IV
Inform local opinion leaders	38	3.35	1.46	3.45	1.15	II	IV
Change accreditation or membership requirements	9	3.25	1.25	3.20	1.24	IV	IV
**6. Leadership & Organization**							
Mandate change	44	4.65	0.59	3.35	1.04	I	I
Ensure managerial support and embedding	74	4.63	0.50	4.00	0.75	I	I
Provide digital equipment and knowledge	85	4.21	1.03	3.84	1.01	I	I
Ensure program is in line with legal frameworks and requirements	88	4.68	0.48	4.00	1.00	I	I
Build service capacity	91	4.32	0.75	3.37	0.96	I	I
Inform employees about the implementation plan	97	3.63	1.01	3.32	0.95	I	II
Involve executive boards	40	3.75	1.33	2.80	1.24	III	III
Obtain formal commitments	47	3.05	1.15	3.20	1.11	IV	II
Change service sites	13	1.65	1.27	1.55	1.10	IV	IV
Revise professional roles	59	2.15	1.09	2.20	1.01	IV	IV
**7. Training**							
Conduct educational outreach visits	16	4.35	0.75	4.15	0.81	I	I
Develop educational materials	29	4.50	0.61	3.85	1.04	I	I
Distribute educational materials	31	4.30	1.03	4.35	0.88	I	I
Provide counseling in working with ambivalence among parents	86	4.37	0.76	4.16	0.76	I	I
Ensure regular program and content updates	94	4.16	0.69	3.89	0.88	I	I
Make training dynamic	43	3.70	0.86	3.45	0.76	I	IV
Create an extended program and treatment manual	96	3.84	0.83	3.74	0.93	I	IV
Conduct educational meetings	15	3.10	1.12	3.10	1.12	IV	IV
**8. Clinician Support & Implementation Counseling**							
Conduct ongoing training	19	4.55	0.76	3.60	0.88	I	I
Develop an implementation glossary	25	4.05	1.15	3.65	1.04	I	I
Provide clinical supervision	53	4.65	0.49	3.75	0.91	I	I
Use train-the-trainer strategies	71	4.10	0.72	3.80	0.89	I	I
Provide training for the entire clinic/service (a generalist model)	75	4.42	0.61	3.63	0.60	I	I
Create resource teams in the service	82	4.11	0.74	4.05	0.71	I	I
Teach practitioners to work according to evidence-based principles	76	3.63	0.96	3.47	0.90	I	II
Create regular mentoring/team meetings	77	3.63	0.83	3.68	0.58	I	II
Provide ongoing consultation	55	3.60	0.99	3.40	1.10	II	IV
Provide local technical assistance	54	3.70	1.03	3.20	1.20	III	IV
Shadow other experts	60	2.90	1.21	2.85	1.09	IV	IV
Use advisory boards and workgroups	64	2.65	1.09	2.55	1.05	IV	IV
Use an implementation advisor	65	2.90	1.33	3.00	1.30	IV	IV
**9. Quality Assurance**							
Recruit, designate, and train for leadership	57	3.80	1.11	3.45	1.00	I	I
Make a plan for treatment referrals	95	3.74	1.10	3.05	1.03	III	III
Ensure program fidelity	84	3.58	0.90	3.16	1.07	IV	II
Create or change credentialing and/or licensure standards	22	3.30	1.03	2.85	1.04	IV	IV
**10. User Involvement & Participation**							
Involve patients/consumers and family members	41	4.30	0.80	3.75	0.85	I	I
Obtain and use patients and family feedback	46	4.70	0.47	3.60	0.88	I	I
Prepare patients to be active participants	50	4.55	0.76	4.10	0.79	I	I
Increase demand	37	3.70	1.26	3.45	0.83	I	II
Intervene with patients to enhance uptake and adherence	39	3.90	1.02	3.30	1.03	I	III
Make cultural adaptations to the program	90	4.26	0.73	3.21	0.85	I	III
Use mass media	69	2.85	1.14	3.40	1.19	II	II
Create an e-learning course for practitioners	79	2.89	1.20	3.37	1.12	II	IV
Develop resource sharing agreements	30	2.95	1.05	2.35	1.09	IV	IV

**Fig. 2:**
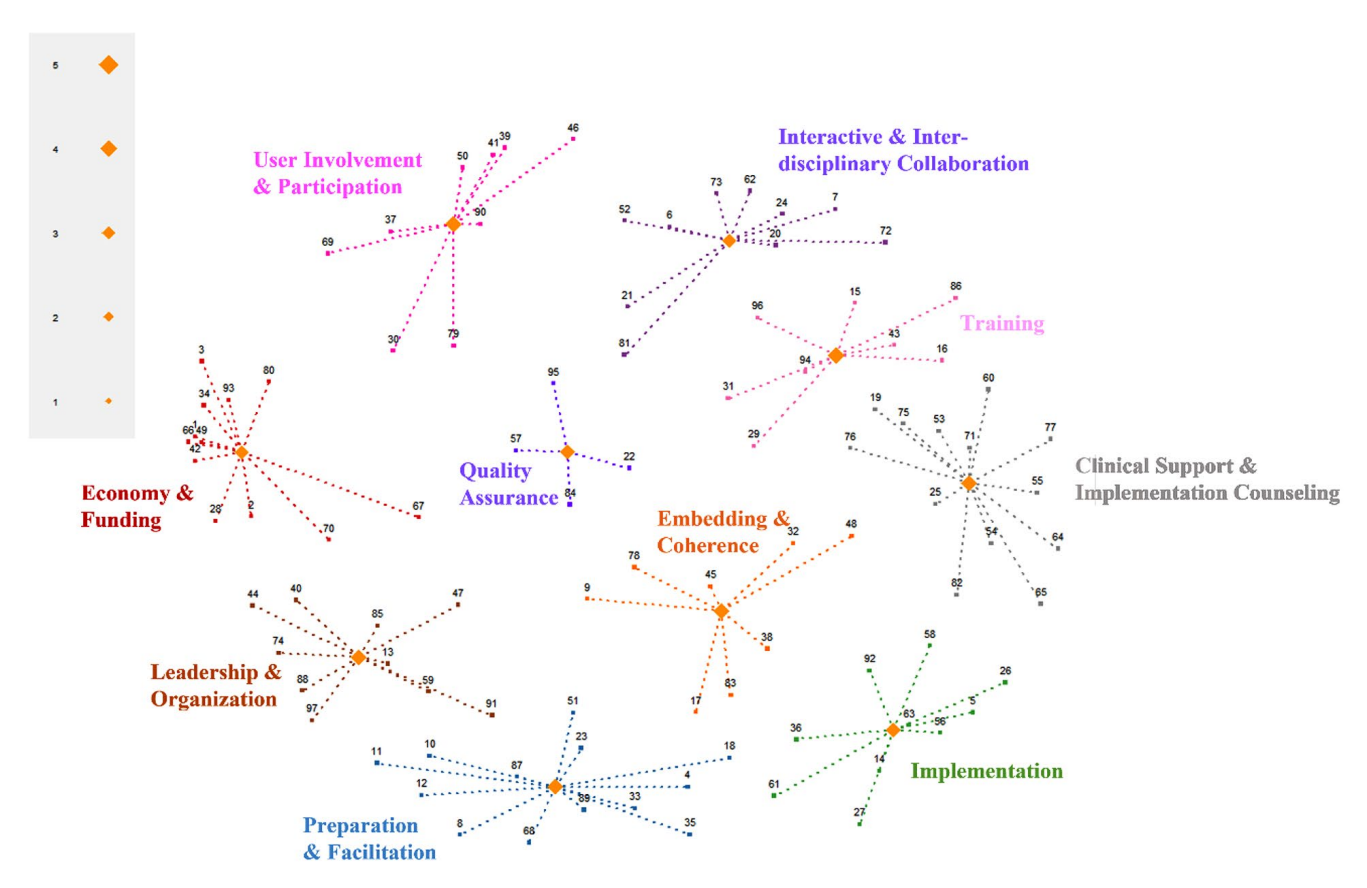
A point and cluster rating map for each cluster by importance (*n* = 20)

Figure 2 presents a point and cluster rating map that visually represents the relationship between the 97 strategies, accompanied by a number for cross-referencing to the strategies in Tables 2 and 3. In general, the closer two strategies are together, the more often they were sorted together (e.g., strategies 1 (access new funding) and 49 (place innovation on fee for service lists) in the *Economy and Funding* cluster; see Fig. 2). Strategies farther apart from each other were less often, if at all, sorted together (e.g., strategies 11 (change physical structure and equipment) and 16 (conduct educational outreach visits) in the *Preparation and Facilitation* and *Training* clusters, respectively). Similarly, clusters near one another are more closely connected than those farther away. Clusters in the middle of the map (i.e., *Quality Assurance* and *Embedding and Coherence*) can be considered to function as a bridge for interaction between other clusters. For example, establishing a coherent individual and collective understanding of a new practice, can make any preparations and training, both more meaningful and thus easier to embed into routine practice.

**Fig. 3:**
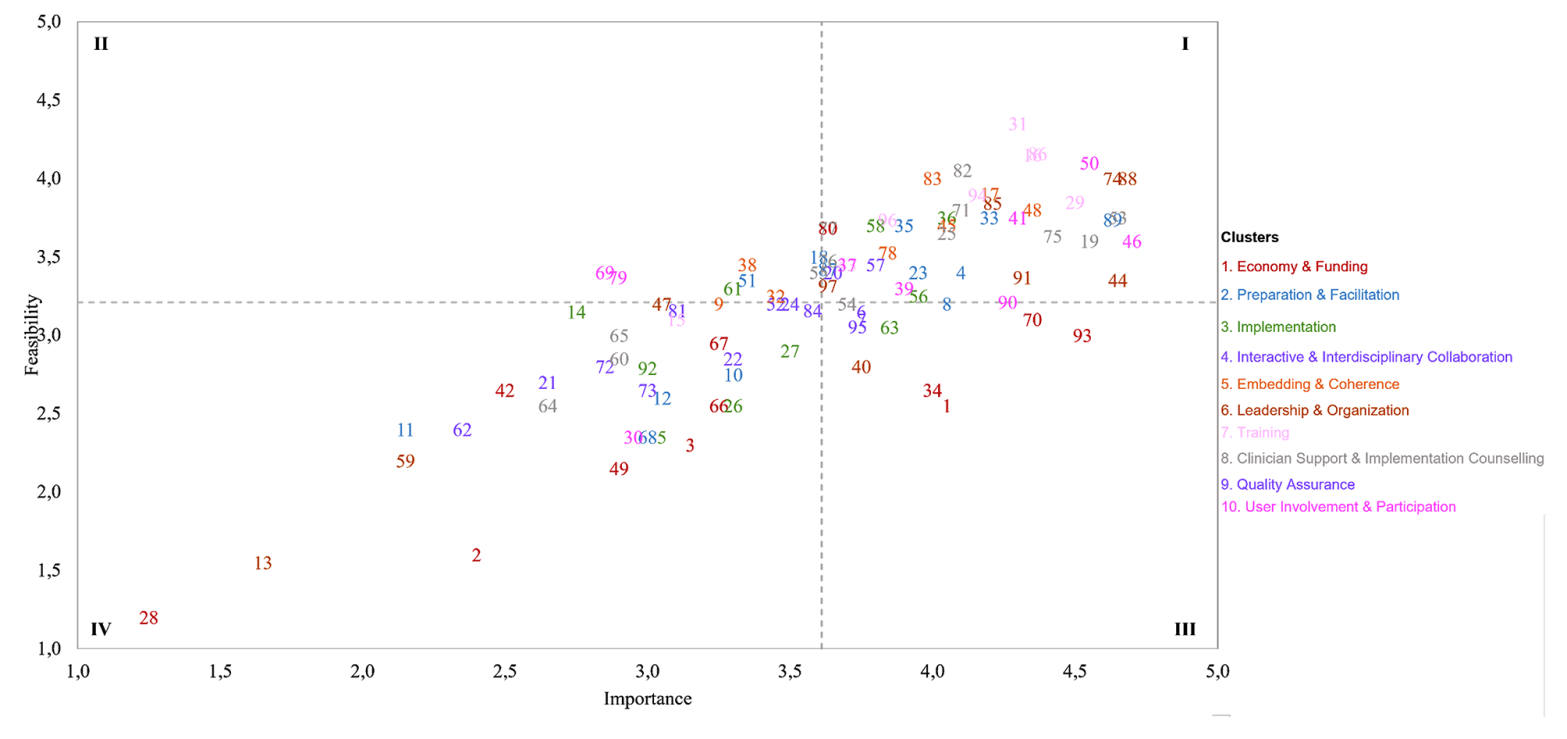
Global go-zone graph for all 97 strategies (*n* = 20)

Figure 3 shows the global Go-Zone graph for each of the 97 strategies. The graph was divided into four quadrants by the average importance (*M* = 3.61, *SD* = 0.70) and feasibility (*M* = 3.21, *SD* = 0.59) ratings. There was a significant correlation between ratings (*r* =.62, *p* =.004), indicating that most strategies fell within quadrants I (*n* = 44, 45.4%) or IV (*n* = 34, 35.1%). The upper right quadrant (I), referred to as the Go-Zone, shows strategies that were rated above average on both importance and feasibility. These strategies were mostly from clusters 2, 5, 6, 7, 8, and 10 (i.e., *Preparation and Facilitation*, *Embedding and Coherence*, *Leadership and Organization*, *Training, Clinician Support and Implementation Counseling*, and *User Involvement and Participation*; see also Tables 2 and 3), and should be prioritized and addressed first in any ensuing implementation plan. Conversely, strategies rated lowest on both importance and feasibility, fell within the lower left quadrant (IV; i.e., the No-Go zone). These were predominantly from clusters 1 to 4 (i.e., *Economy and Funding*, *Preparation and Facilitation*, *Implementation*, and *Interactive and Interdisciplinary Collaboration*; see also Tables 2 and 3). Only a few strategies were rated relatively important (upper left quadrant III; *n* = 11, 11.3%) or feasible (lower right quadrant II; *n* = 8, 8.2%). Table 3 also shows the Go-Zone quadrants for each cluster independently. Although most of the strategies (*n* = 66, 68.0%) remained in the same quadrant as in the global Go-Zone analysis, 31 (32.0%) strategies were classified into another quadrant in the per cluster analysis. Changes in quadrants among strategies between the global and per cluster analyses occurred across all clusters, but mainly in *Economy and Funding* (*n* = 7; 58.3%) and *Interactive and Interdisciplinary Collaboration* (*n* = 5, 50.0%).

**Fig. 4:**
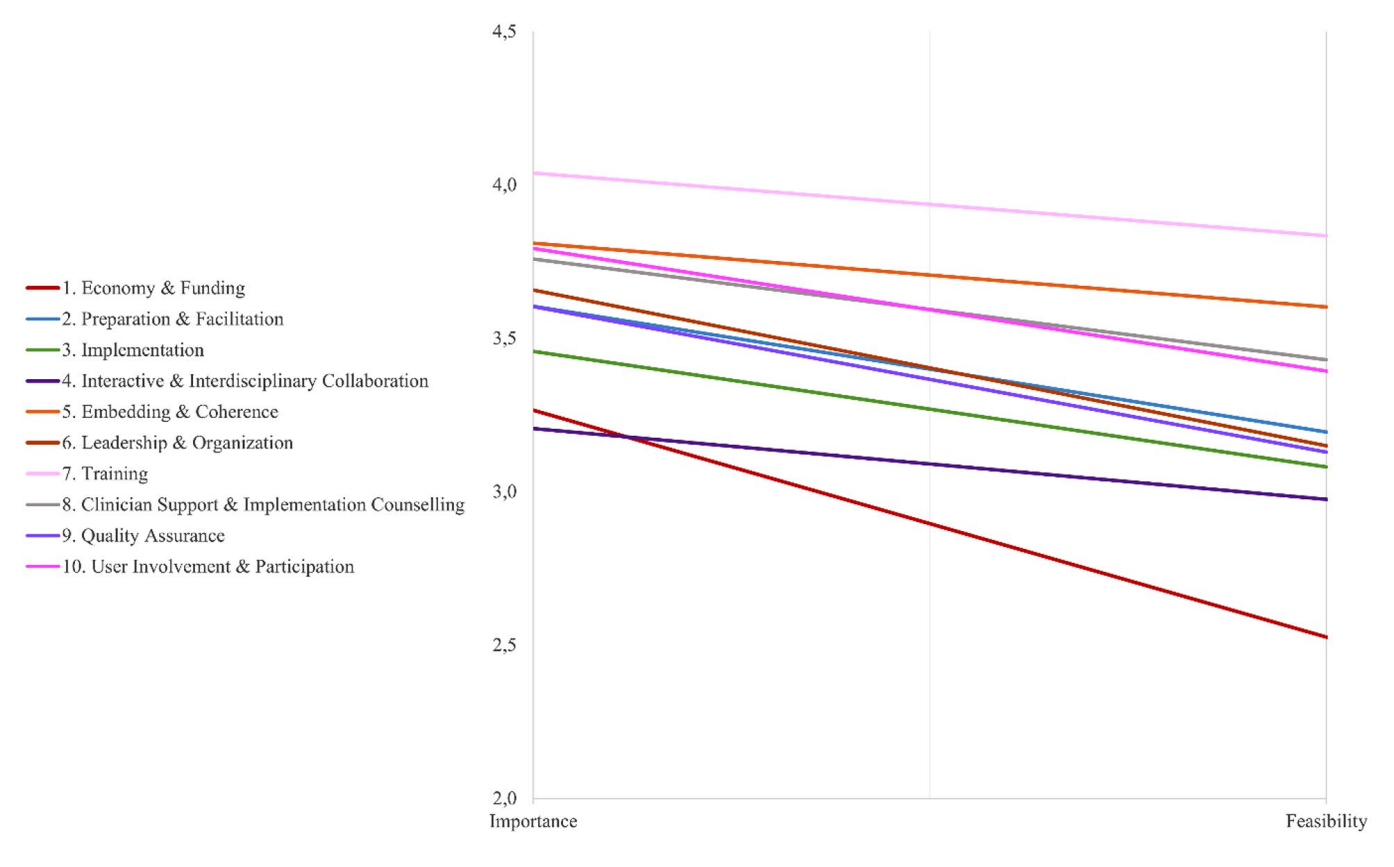
A laddergram comparing the average cluster ratings (*n* = 20)

Figure 4 compares the average importance and feasibility ratings of strategies at the cluster level. It shows that all clusters were judged relatively important, but also consistently more difficult to accomplish. *Training* was considered the most important and feasible, while *Economy and funding* had the greatest mean difference between importance and feasibility ratings and was perceived as least feasible. An analysis of variance revealed statistically significant differences in importance (F (9, 1 906) = 8.91, *p* <.001) and feasibility (F (9, 1 906) = 21.69, *p* <.001) between two or more clusters. Table 4 includes significant differences from Tukey’s test for multiple comparisons of all possible pairs. Most notably, differences show that *Economy and Funding* and *Interactive and Interdisciplinary Collaboration* were rated significantly less important than most other clusters (all *p*s < 0.05). In terms of feasibility, *Training* was perceived as more applicable than all clusters, except *Embedding and Coherence*, while *Economy and Funding* was considered harder to accomplish than all other clusters (all *p*s < 0.05).

**Table 4:** Multiple comparisons of mean differences in importance and feasibility with 95% family-wise confidence level (CI)

Variable	Clusters	Cluster Comparisons	Diff.	95% CI	Adjusted *p-value*
Importance	2–1	Preparation & Facilitation– Economy & Funding	0.34	0.00–0.68	0.046
	5–1	Embedding & Coherence– Economy & Funding	0.55	0.16–0.93	< 0.001
	6–1	Leadership & Organization– Economy & Funding	0.39	0.03–0.75	0.022
	7–1	Training– Economy & Funding	0.77	0.39–1.16	< 0.001
	8–1	Clinician Support & Implementation Counselling– Economy & Funding	0.49	0.16–0.83	< 0.001
	10–1	User Involvement & Participation– Economy & Funding	0.53	0.16–0.90	< 0.001
	4–2	Interactive & Interdisciplinary Collaboration– Preparation & Facilitation	-0.40	-0.75–-0.04	0.013
	7–2	Training– Preparation & Facilitation	0.43	0.05–0.81	0.011
	7–3	Training– Implementation	0.58	0.18–0.98	< 0.001
	5–4	Embedding & Coherence– Interactive & Interdisciplinary Collaboration	0.60	0.20–1.00	< 0.001
	6–4	Leadership & Organization– Interactive & Interdisciplinary Collaboration	0.45	0.07–0.83	0.006
	7–4	Training– Interactive & Interdisciplinary Collaboration	0.83	0.43–1.23	< 0.001
	8–4	Clinician Support & Implementation Counselling– Interactive & Interdisciplinary Collaboration	0.55	0.20–0.91	< 0.001
	10–4	User Involvement & Participation– Interactive & Interdisciplinary Collaboration	0.59	0.20–0.97	< 0.001
Feasibility	2–1	Preparation & Facilitation– Economy & Funding	0.67	0.36–0.98	< 0.001
	3–1	Implementation– Economy & Funding	0.56	0.22–0.89	< 0.001
	4–1	Interactive & Interdisciplinary Collaboration– Economy & Funding	0.45	0.12–0.78	0.001
	5–1	Embedding & Coherence– Economy & Funding	1.08	0.72–1.43	< 0.001
	6–1	Leadership & Organization– Economy & Funding	0.62	0.29–0.96	< 0.001
	7–1	Training– Economy & Funding	1.31	0.95–1.66	< 0.001
	8–1	Clinician Support & Implementation Counselling– Economy & Funding	0.90	0.59–1.21	< 0.001
	9–1	Quality Assurance– Economy & Funding	0.60	0.15–1.05	0.001
	10–1	User Involvement & Participation– Economy & Funding	0.87	0.53–1.21	< 0.001
	5–2	Embedding & Coherence– Preparation & Facilitation	0.41	0.06–0.76	0.008
	7–2	Training– Preparation & Facilitation	0.64	0.29–0.99	< 0.001
	5–3	Embedding & Coherence– Implementation	0.52	0.15–0.89	< 0.001
	7–3	Training– Implementation	0.75	0.39–1.12	< 0.001
	8–3	Clinician Support & Implementation Counselling– Implementation	0.35	0.02–0.67	0.024
	5–4	Embedding & Coherence– Interactive & Interdisciplinary Collaboration	0.63	0.26–0.99	< 0.001
	7–4	Training– Interactive & Interdisciplinary Collaboration	0.86	0.49–1.23	< 0.001
	8–4	Clinician Support & Implementation Counselling– Interactive & Interdisciplinary Collaboration	0.45	0.13–0.78	< 0.001
	10–4	User Involvement & Participation– Interactive & Interdisciplinary Collaboration	0.42	0.06–0.77	0.008
	6–5	Leadership & Organization– Embedding & Coherence	-0.45	-0.82–-0.08	0.004
	7–6	Training– Leadership & Organization	0.69	0.32–1.06	< 0.001
	8–7	Clinician Support & Implementation Counselling– Training	-0.40	-0.75–-0.06	0.009
	9–7	Quality Assurance– Training	-0.71	-1.18–-0.23	< 0.001
	10–7	User Involvement & Participation– Training	-0.44	-0.82–-0.06	0.008

## Discussion

We used GCM to identify strategies for the implementation of a guided IMI for infant sleep problems in well-baby and community mental health clinics. We identified 10 clusters of strategies, of which *Training*, *Embedding and Coherence*, *User Involvement and Participation*, and *Clinician Support and Implementation Counseling* were rated as most important and feasible. In contrast, *Economy and Funding* and *Interactive and Interdisciplinary Collaboration* were rated as least important and feasible. There was a positive linear correlation between the importance and feasibility ratings. Therefore, more strategies from the most important and feasible clusters fell into the Go-Zone quadrant, while more strategies from the least important and feasible clusters fell into the No-Go quadrant. Reflecting on data saturation, the study team found that the discussions added no major changes to the interpretation of results across groups. This likely reflected the narrow study aim, relevance/adequacy of the sample, and the applied use of a specific taxonomy and methodology providing a clear and structured dialogue between researchers and participants. In what follows, we discuss the most prominent results and interpretation of findings from discussions with participants. The references to strategies are numbered in parentheses for cross-referencing to the cluster map (Fig. 1) and Tables 2 and 3.

Overall, the results resonated with the participants. The stress value was acceptable, indicating that there is a structure to the data. Further validation of these findings can be found in Waltz and colleagues who conducted a GCM-study using the ERIC-taxonomy with implementation experts [48]. Although our study included clinical staff and additional strategies, participants conceptualized strategies in similar ways. Apart from the slightly different labels, Waltz and colleagues also identified clusters related to financial strategies, training, engaging users, collaboration with stakeholders, and supporting clinicians [48]. However, how participants sorted strategies within clusters, varied greatly. For example, we found that participants conceptualized ongoing training (19) and consultation (55) as *Clinical Support and Implementation Counseling*, rather than training and educating stakeholders. This may be due to the different groups of participants in the studies but may also show how the conceptualization of strategies can vary across cultures and contexts.

In discussing the findings, the participants recognized that IMIs do have setup, operation, and maintenance costs [21], but that they, as clinical staff, rarely have opportunities to influence the funding of their clinic. Therefore, *Economy and Funding* should not be interpreted as unimportant but must be taken care of at higher system levels (e.g., municipal or government funding), as successful examples of IMIs in routine practice have taught us [22]. Thus, it makes sense that *Leadership and Organization* were close to *Economy and Funding* on the cluster map. Participants were more concerned about learning to administer the program and any counseling methods [42], but did not consider that *Training* in IMIs needed to be extensive. IMIs do not require the same level of competency or skills training as face-to-face methods, as parents carry out much of the intervention themselves. However, the participants were clear about the need for active involvement in *Training* by participating in group work, testing, and administration of the program.

*Interactive and Interdisciplinary Collaboration* was rated less important and feasible compared to many clusters. During the discussions, it became clear that some strategies in this cluster were considered not currently relevant (e.g., 62), useful but not necessary (e.g., 72), or the responsibilities of other stakeholders (e.g., 24 and 73). It was more important to create structures for learning collaboratives (20) where clinicians could meet regularly to learn and share experiences. Further building of coalitions (6) and network weaving (52) could be considered important, but mainly to embed the IMI into routine care. *Embedding* new practices is made possible by an understanding of their meaning, uses, and utility. It requires a coherent set of beliefs and behaviors that define and organize the work, and that are seen as meaningful and different from other practices (78) [49]. In this sense, participants were surprised that informing local opinion leaders (38) was not in the Go-Zone, as they considered it essential, and should include key managers, clinicians, and administrative staff.

Regardless of clustering, certain strategies fell in the No-Go zone because they are rarely used in public healthcare in Norway (e.g., 2 (alter incentive/allowance structures) and 59 (revise professional roles)) or may be experienced as unpleasant and stressful (e.g., 26 and 27 (tools for quality monitoring), and 81 (certification schemes)). Participants agreed that purveyors must set certain quality requirements for the delivery of IMIs, but this can be achieved in other ways than through licensing standards (22) or certification schemes (81). *Quality Assurance* and evaluative strategies in general require a high level of psychological safety [50]. Therefore, many were reluctant to such strategies and are not used to being monitored or measured in performance. *Quality Assurance* and evaluative strategies were also compared to established initiatives such as specialist breastfeeding centers, which use formal quality requirements [51]. According to the participants, it must be acknowledged that evaluative strategies only provide a snapshot of the current situation and take time away from families in an already busy work schedule.

Finally, participants acknowledged the distances and spatial relationships between strategies in the cluster map (Fig. 2). Strategies within clusters spread in different spatial directions and some were farther from the center of the cluster than others, approaching neighboring clusters such as conducting local consensus discussion (17) in *Embedding and Coherence* and needs assessment (18) in *Preparation and Facilitation*. Participants explained that some strategies, such as those related to *Training* and *Economy and Funding*, were easier to sort than others, and not all the strategies were equally clear or understandable. Several mentioned that strategies could have been sorted in multiple ways or placed in several piles. They perceived that there were subtle differences between several strategies and even considered them interchangeable (e.g., reexamining the implementation (56) and audit and feedback (5)). Furthermore, the participants pointed out that the role they had in the clinic (leader vs. clinician) and professional background (PHN vs. psychologist) could also have impacted how they sorted and rated the strategies.

### Strengths and limitations

GCM is an efficient and engaging method to obtain insights on a topic. It is less resource intensive than interviews, but there are also limited possibilities to probe and explore new concepts and may not provide sufficient in-depth data [52]. However, one of its strengths is the mixed methods approach. This became evident in the discussions where participants attributed less value to *Economy and Funding* but recognized that the ratings reflected their opportunities to influence the funding of a clinic, more than its actual importance.

For practical reasons, it was not possible to discuss the updated maps that included the strategies from the brainstorming. However, the discussions of maps based on the ERIC-taxonomy were audio-recorded, summarized, and reviewed against the final maps. Most clusters and sorting of strategies remained the same, but discussions may have given participants more time to reflect and fine-tune their sorting to truly represent their views.

It is important to note that although each cluster is unique, there are overlapping ideas between them. Participants expressed that certain strategies were more difficult to sort and that they could have sorted the strategies in multiple ways or placed certain strategies in several piles. However, in GCM, a strategy or statement can only be placed in one pile. Thus, overlaps are inevitable and common; also, because participants are instructed to sort strategies in a way that makes sense to them, without being guided by any theory or logic. Although this may be a limitation, it can also be considered a strength as it highlights challenging, ambivalent, or even contradictory concepts or ideas that may have important implications for, in our case, the implementation of the IMI.

Although the study included more than the recommended number of participants for GCM [43], subgroups of interest became unbalanced and small due to the modest sample size. For such reasons, we did not compare clustering or ratings between primary care services (i.e., well-baby vs. community clinics), professions (i.e., PHNs vs. psychologists), or roles (e.g., leaders and clinicians). This could have provided a more nuanced understanding of the implementation strategies. It could also be argued that the heterogeneity among participants more closely resembles the real-world setting in which the IMI will be implemented and thus has captured important variations in their responses. Taken together, the overlapping strategies and heterogeneity among our group of participants may reflect the modest overall consistency (i.e., split-half reliability) of the sorting task. Yet, the stress value indicated that the relationship between the data, similarity matrix, and distances on the map, was acceptable and that there is an underlying structure in the data.

## Conclusions

GCM made it possible to efficiently examine the perspectives of the well-baby clinics and community clinics on complex issues, and to acquire specific knowledge to allow for the planning and prioritization of implementation strategies. *Training*, *Embedding and Coherence*, *User Involvement and Participation*, and *Clinician Support and Implementation Counseling* were identified as the most important and applicable areas for implementation. In contrast, *Economy and Funding* and *Interactive and Interdisciplinary Collaboration* were rated as least important and feasible, although they should not be ignored but taken care of for sustainable implementation. Cluster-level Go-Zone analyzes and the discussions of the findings with participants may help identify which strategies within clusters to target. These results suggest areas of priority for the implementation of IMIs related to infant sleep problems such as *Sleep Well, Little Sweetheart*, and potentially other practices in primary care for parents with young children.

### Electronic supplementary material

Below is the link to the electronic supplementary material.

Supplementary Material 1

## Data Availability

Data are stored at the Regional Center for Child and Adolescent Mental Health, Eastern and Southern Norway, but cannot be shared publicly as consent for publication of the dataset was not obtained. Requests to access the datasets should be directed to Filip Drozd: filip.drozd@r-bup.no.

## Abbreviations

ERIC-taxonomy: Expert Recommendations for Implementing Change-taxonomy
HPs: Health professionals
IMIs: Internet- and mobile-based interventions
GCM: Group concept mapping
PHNs: Public health nurses
