# Supplementary material for: Identifying and developing strategies for implementation of a guided internet- and mobile-based infant sleep intervention in well-baby and community mental health clinics using group concept mapping
Source: BMC Health Serv Res. 2024 Feb 7;24:175. doi: 10.1186/s12913-024-10632-w (PMC10851561; doi:10.1186/s12913-024-10632-w)
Supplement: Supplementary file 1 — Supplementary Material 1 [file 12913_2024_10632_MOESM1_ESM.pptx]

## Slide 1
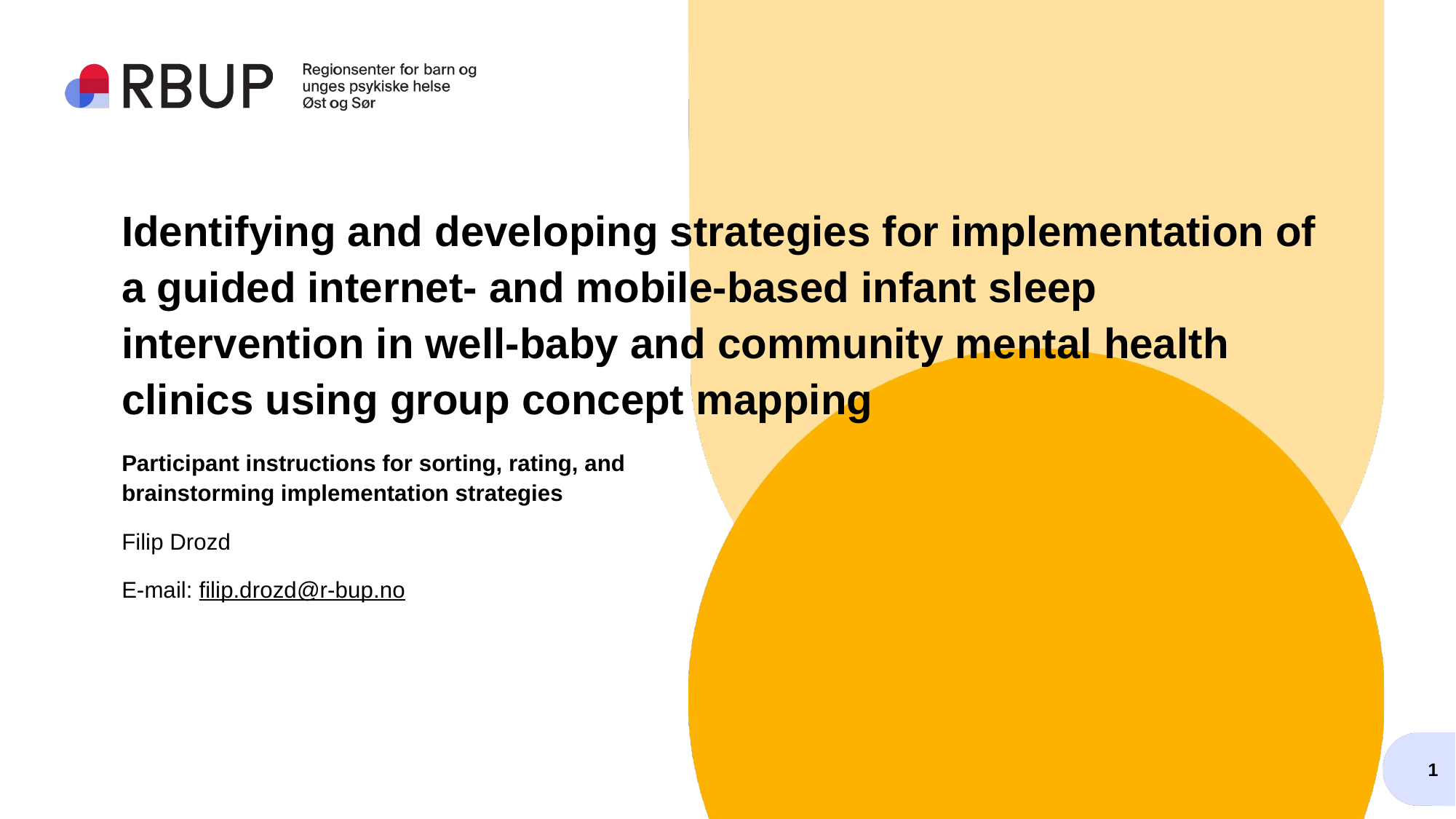

# Identifying and developing strategies for implementation of a guided internet- and mobile-based infant sleep intervention in well-baby and community mental health clinics using group concept mapping
Participant instructions for sorting, rating, and brainstorming implementation strategies
Filip Drozd
E-mail: filip.drozd@r-bup.no
1

## Slide 2
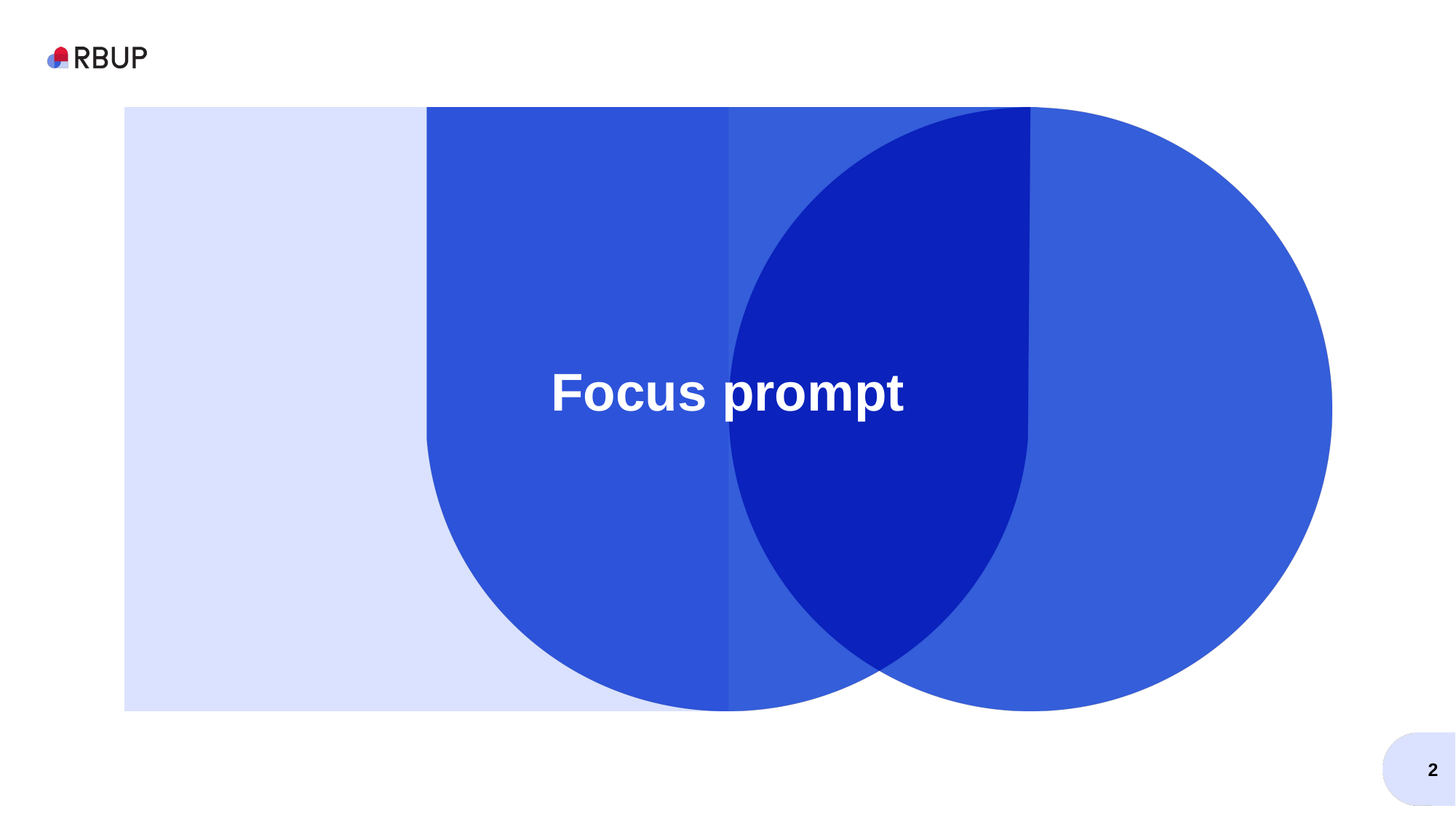

# Focus prompt
2

## Slide 3
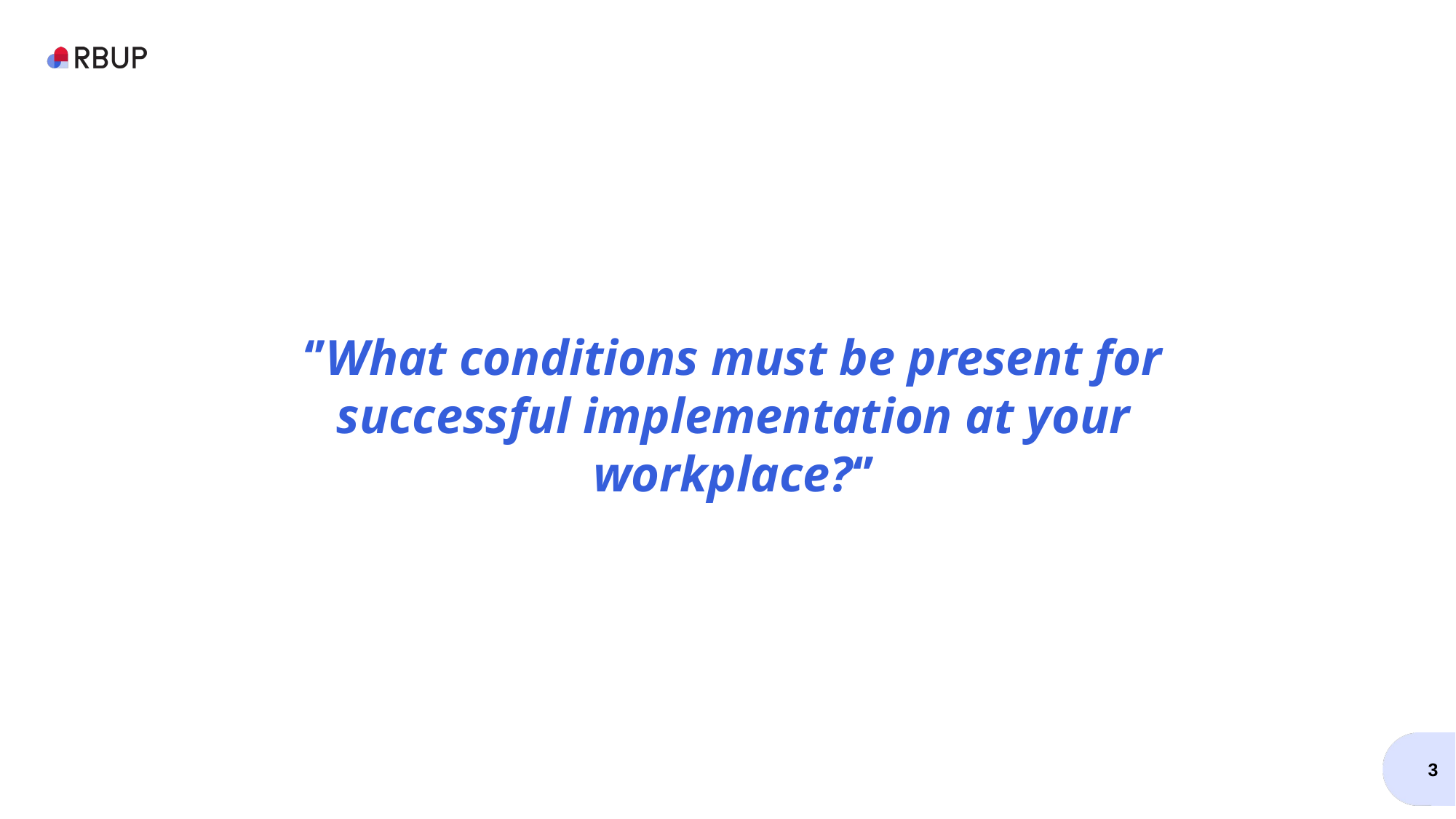

‘’What conditions must be present for successful implementation at your workplace?‘’
3

## Slide 4
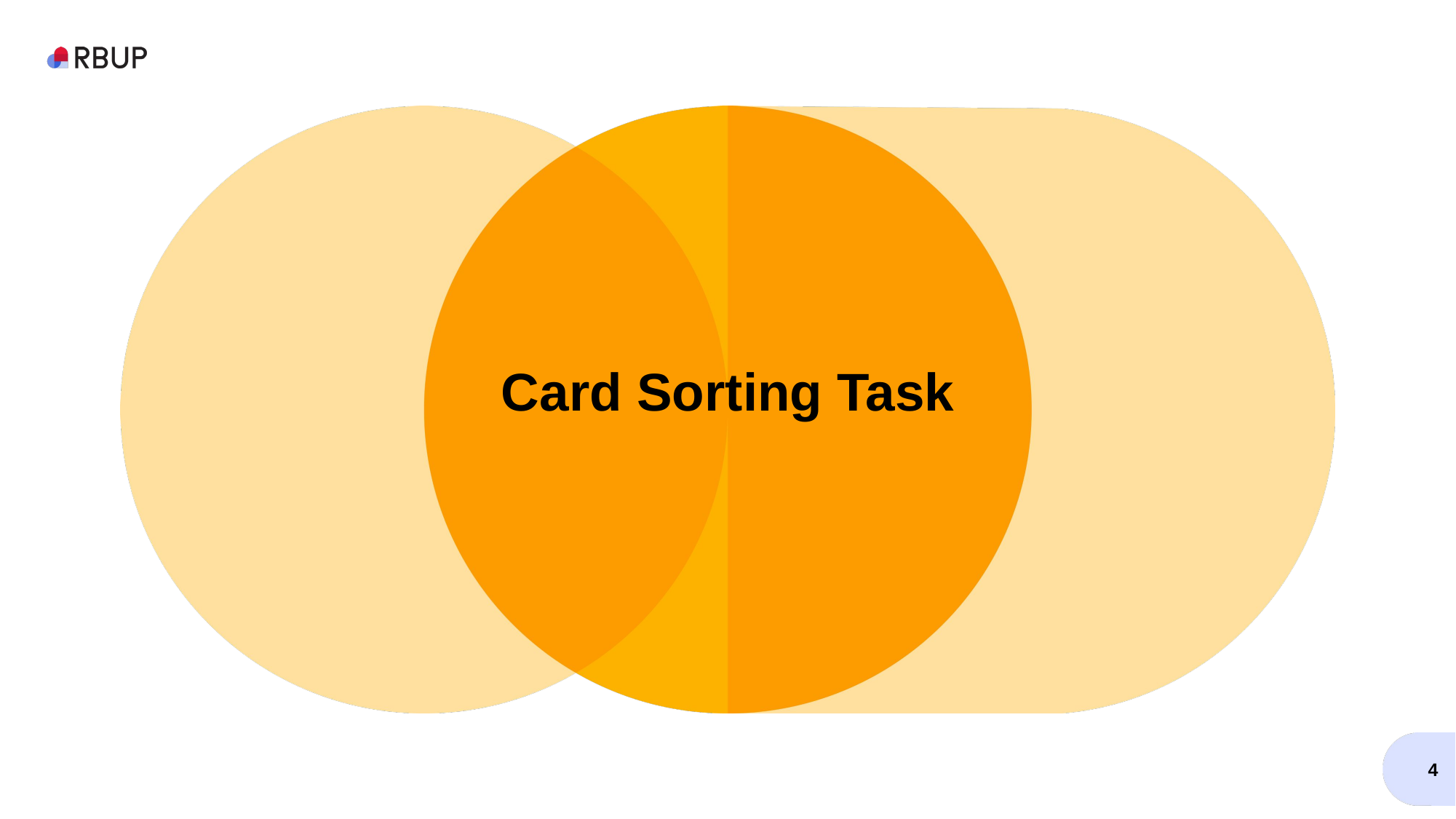

# Card Sorting Task
4

## Slide 5
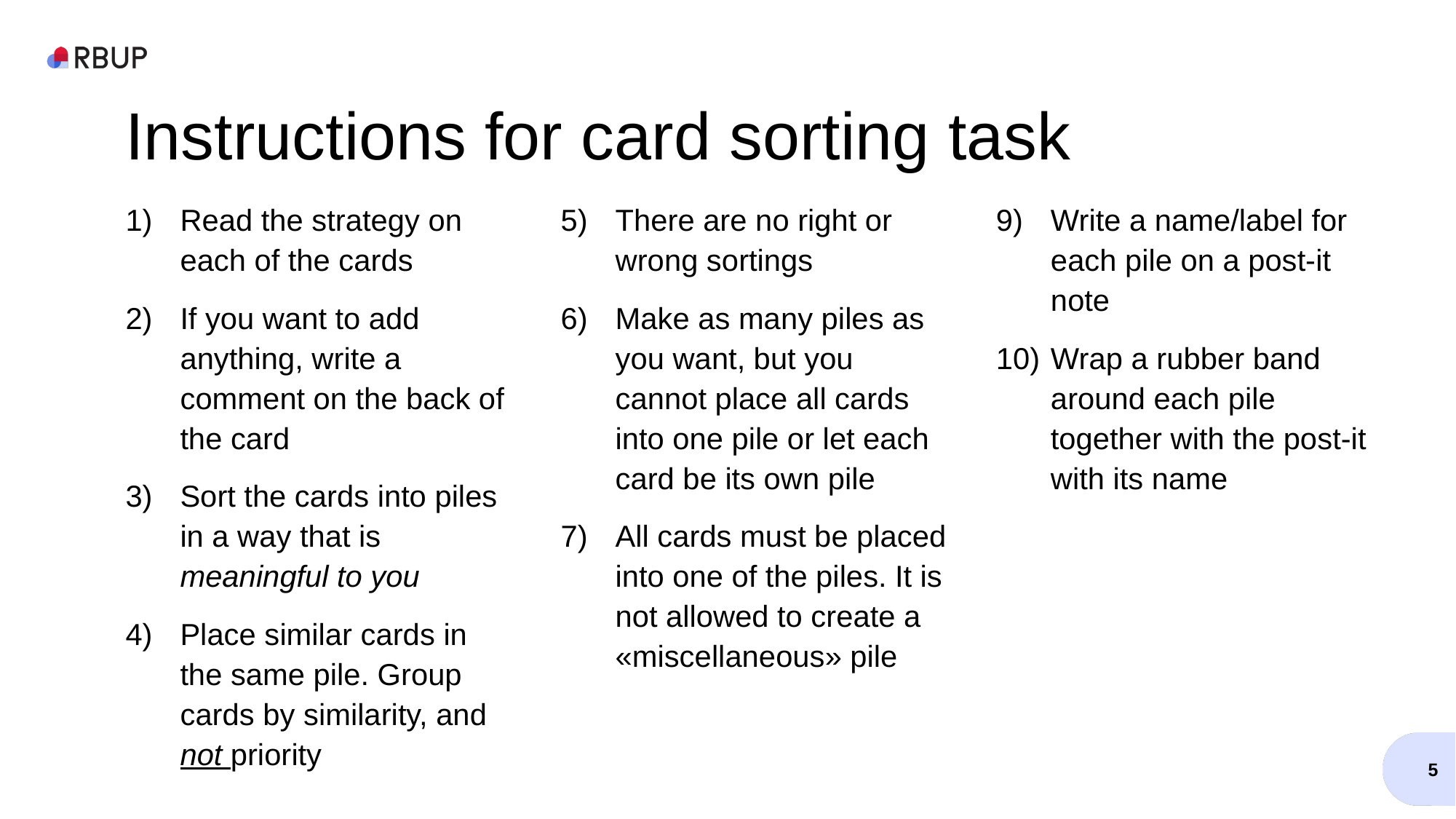

# Instructions for card sorting task
Read the strategy on each of the cards
If you want to add anything, write a comment on the back of the card
Sort the cards into piles in a way that is meaningful to you
Place similar cards in the same pile. Group cards by similarity, and not priority
There are no right or wrong sortings
Make as many piles as you want, but you cannot place all cards into one pile or let each card be its own pile
All cards must be placed into one of the piles. It is not allowed to create a «miscellaneous» pile
Write a name/label for each pile on a post-it note
Wrap a rubber band around each pile together with the post-it with its name
5

## Slide 6
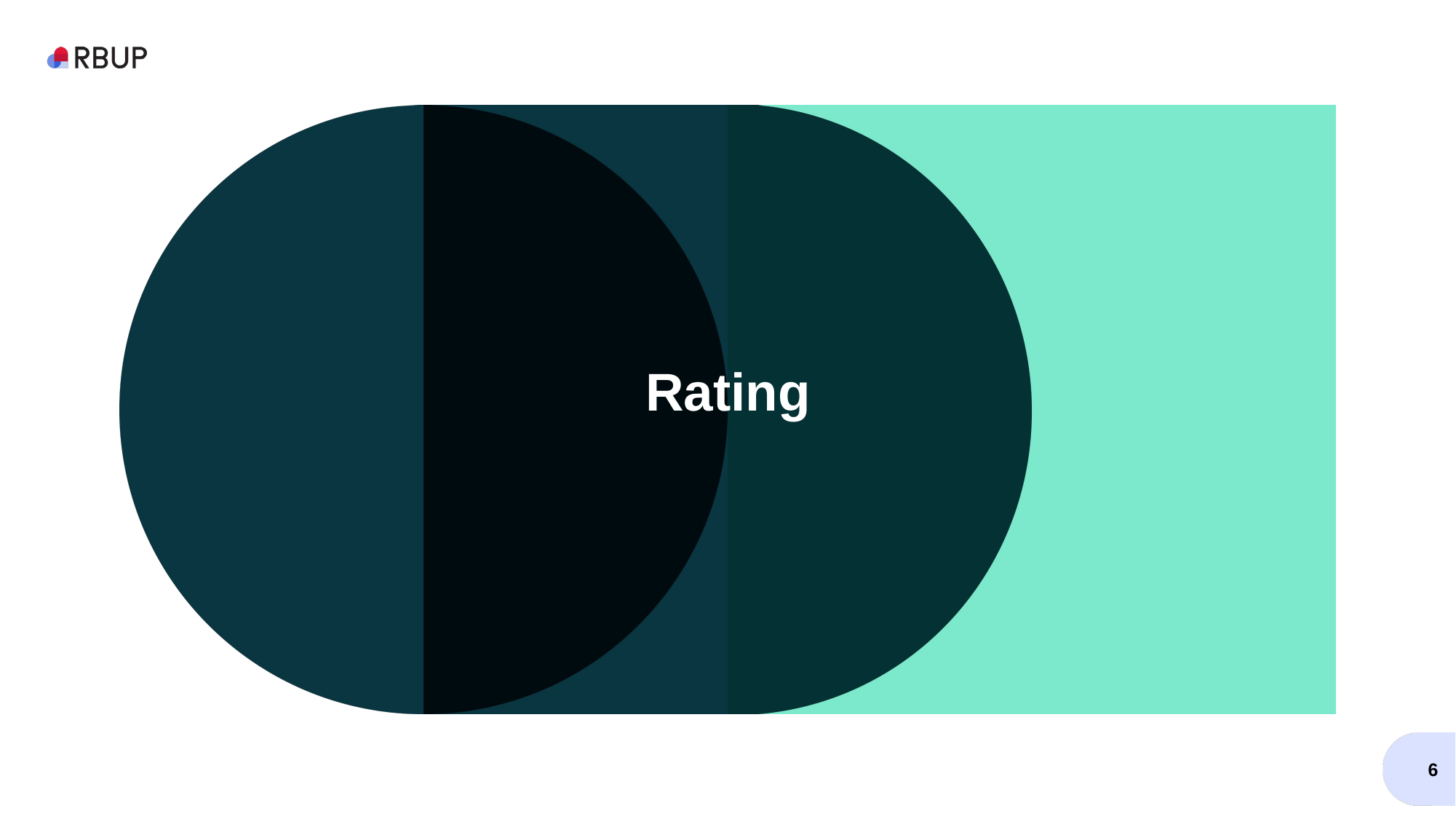

# Rating
6

## Slide 7
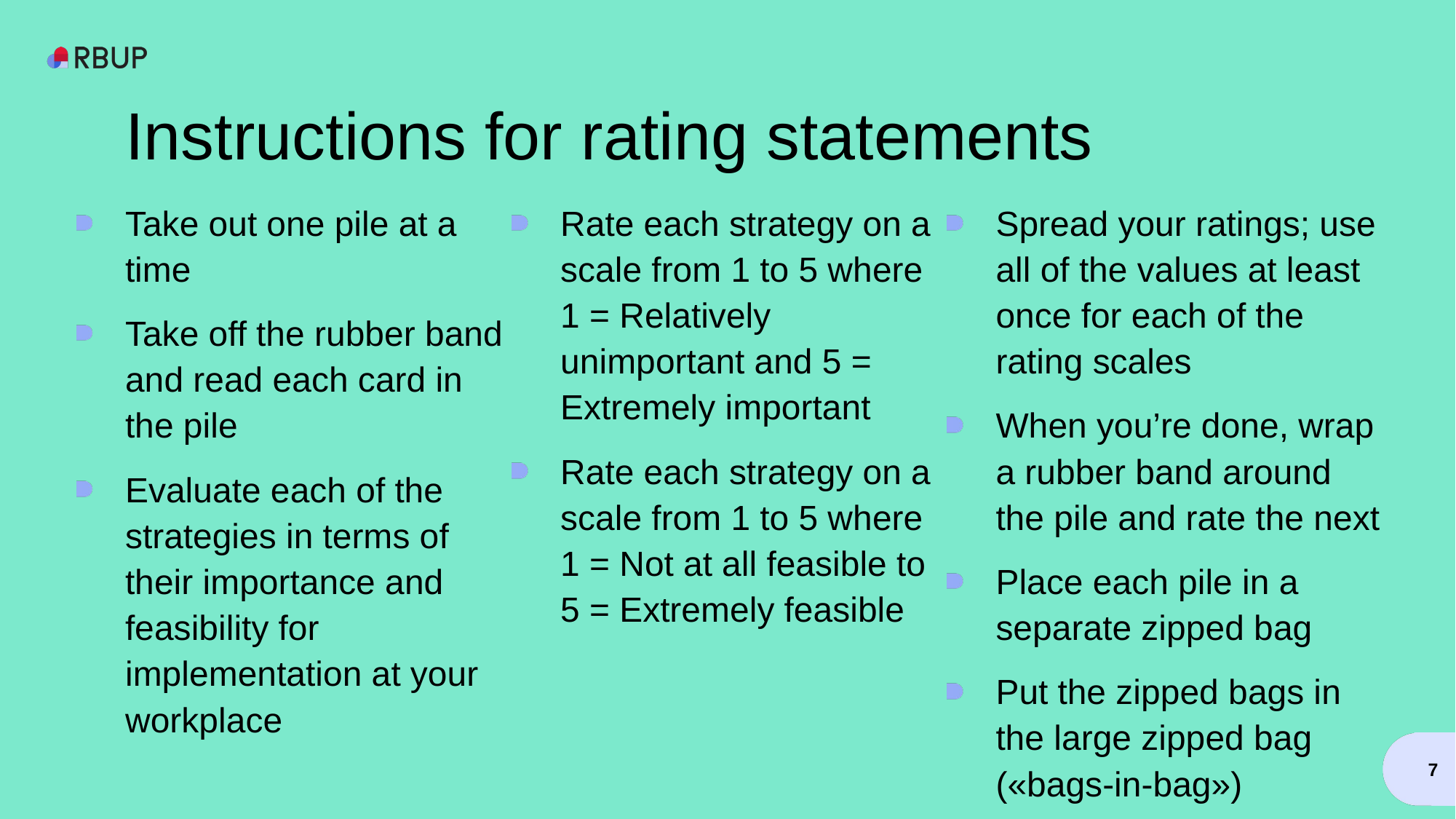

# Instructions for rating statements
Take out one pile at a time
Take off the rubber band and read each card in the pile
Evaluate each of the strategies in terms of their importance and feasibility for implementation at your workplace
Rate each strategy on a scale from 1 to 5 where 1 = Relatively unimportant and 5 = Extremely important
Rate each strategy on a scale from 1 to 5 where 1 = Not at all feasible to 5 = Extremely feasible
Spread your ratings; use all of the values at least once for each of the rating scales
When you’re done, wrap a rubber band around the pile and rate the next
Place each pile in a separate zipped bag
Put the zipped bags in the large zipped bag («bags-in-bag»)
7

## Slide 8
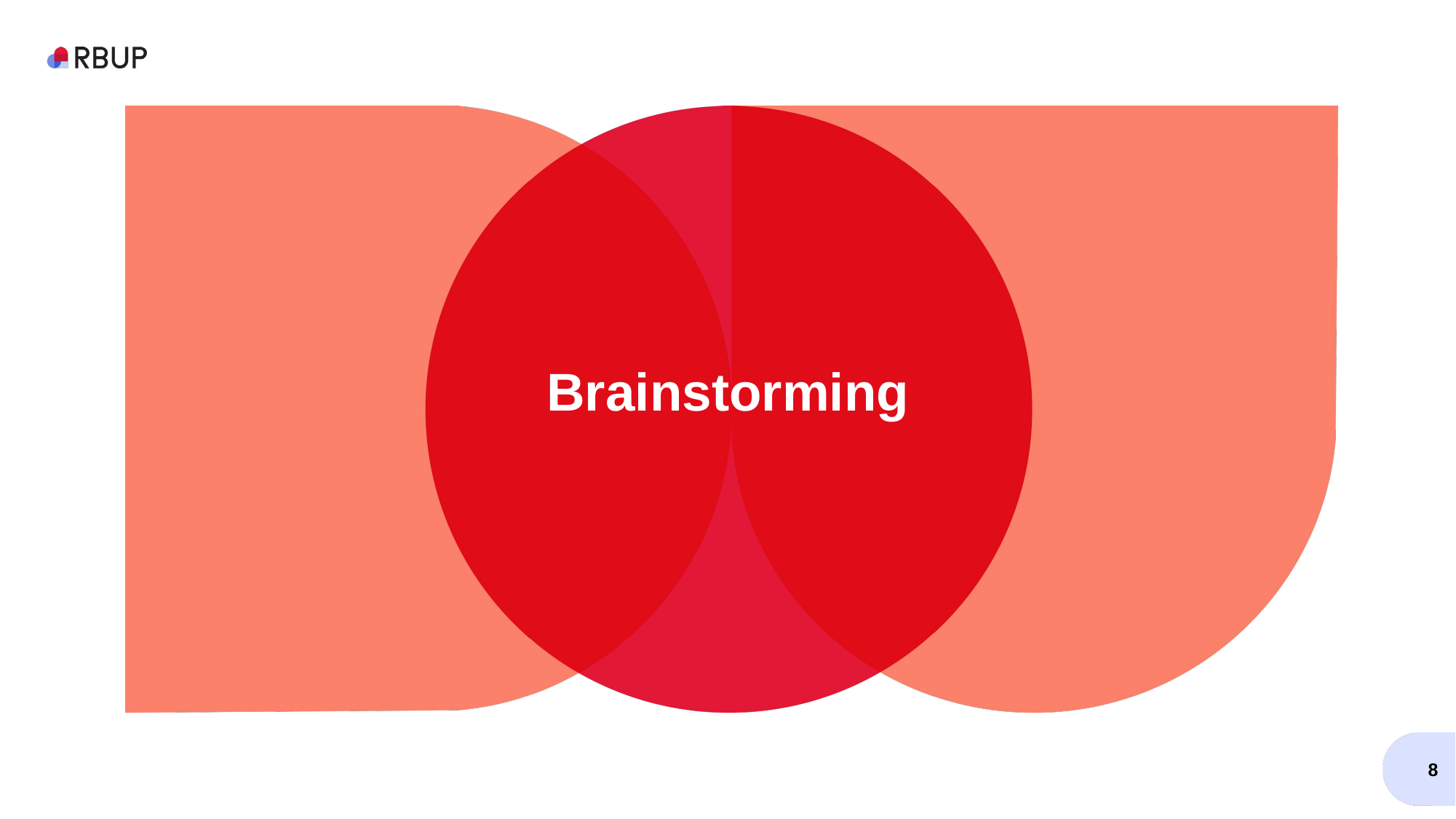

# Brainstorming
8

## Slide 9
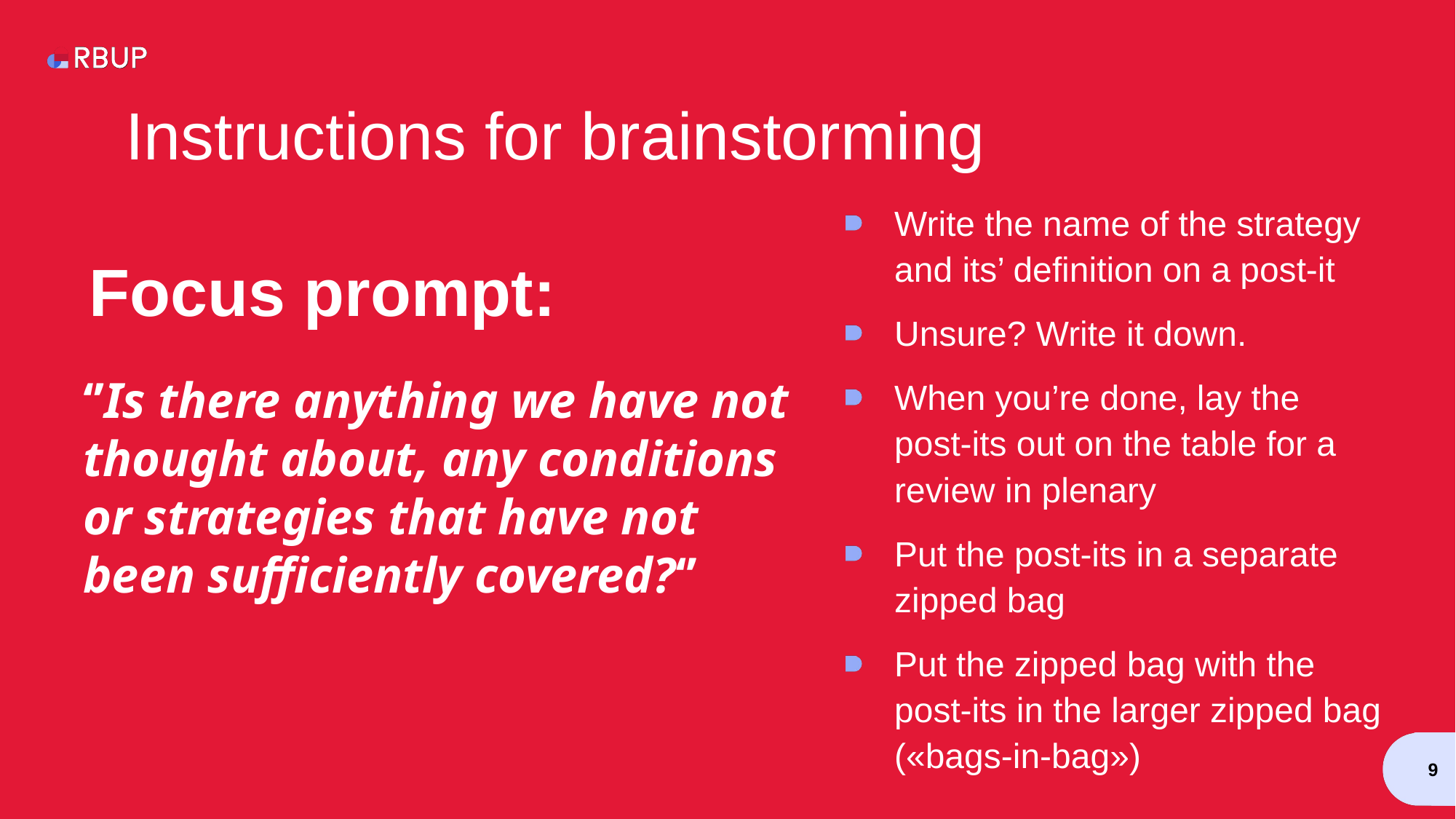

# Instructions for brainstorming
Write the name of the strategy and its’ definition on a post-it
Unsure? Write it down.
When you’re done, lay the post-its out on the table for a review in plenary
Put the post-its in a separate zipped bag
Put the zipped bag with the post-its in the larger zipped bag («bags-in-bag»)
Focus prompt:
‘’Is there anything we have not thought about, any conditions or strategies that have not been sufficiently covered?‘’
9

## Slide 10
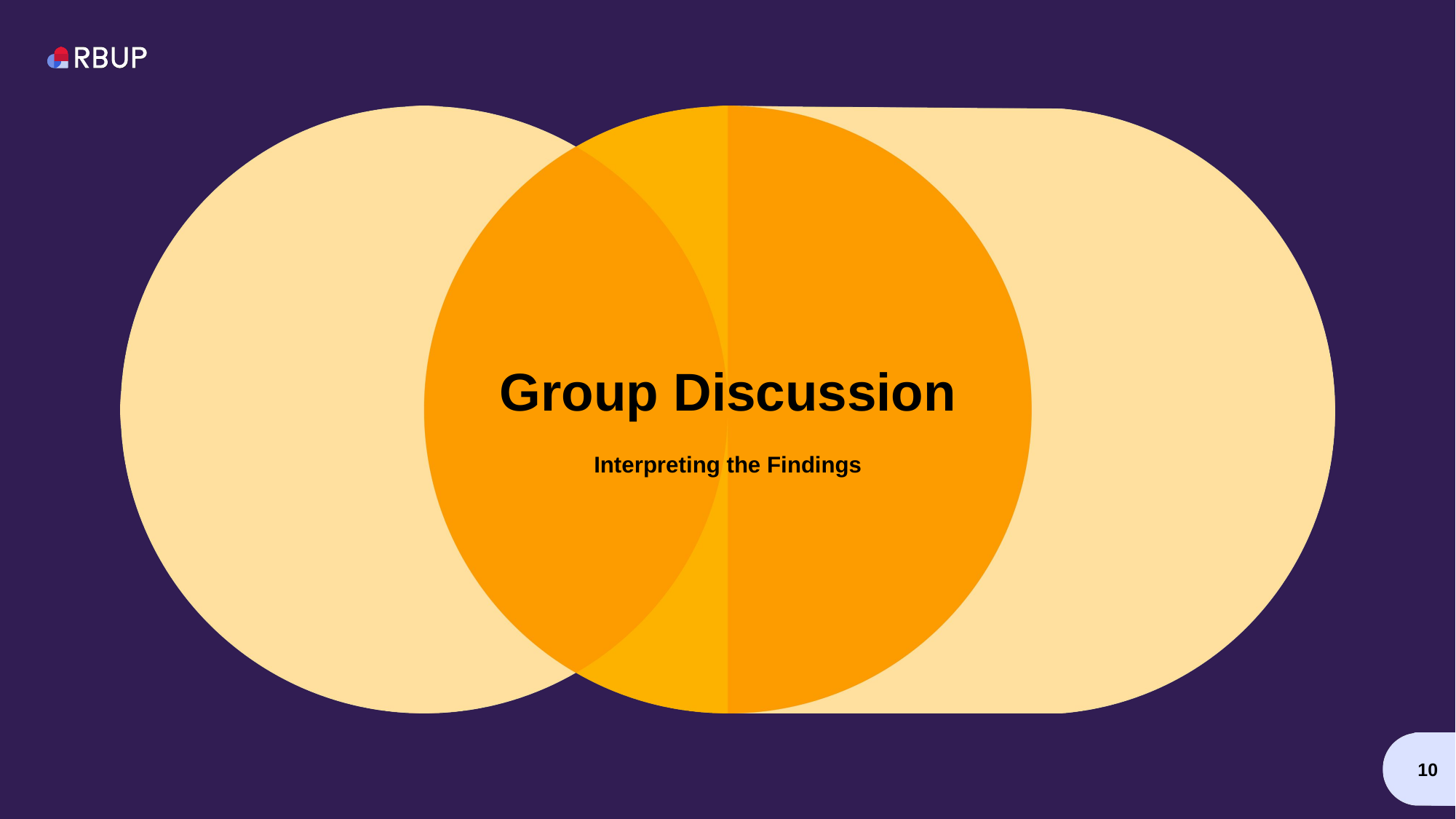

# Group Discussion
Interpreting the Findings
10

## Slide 11
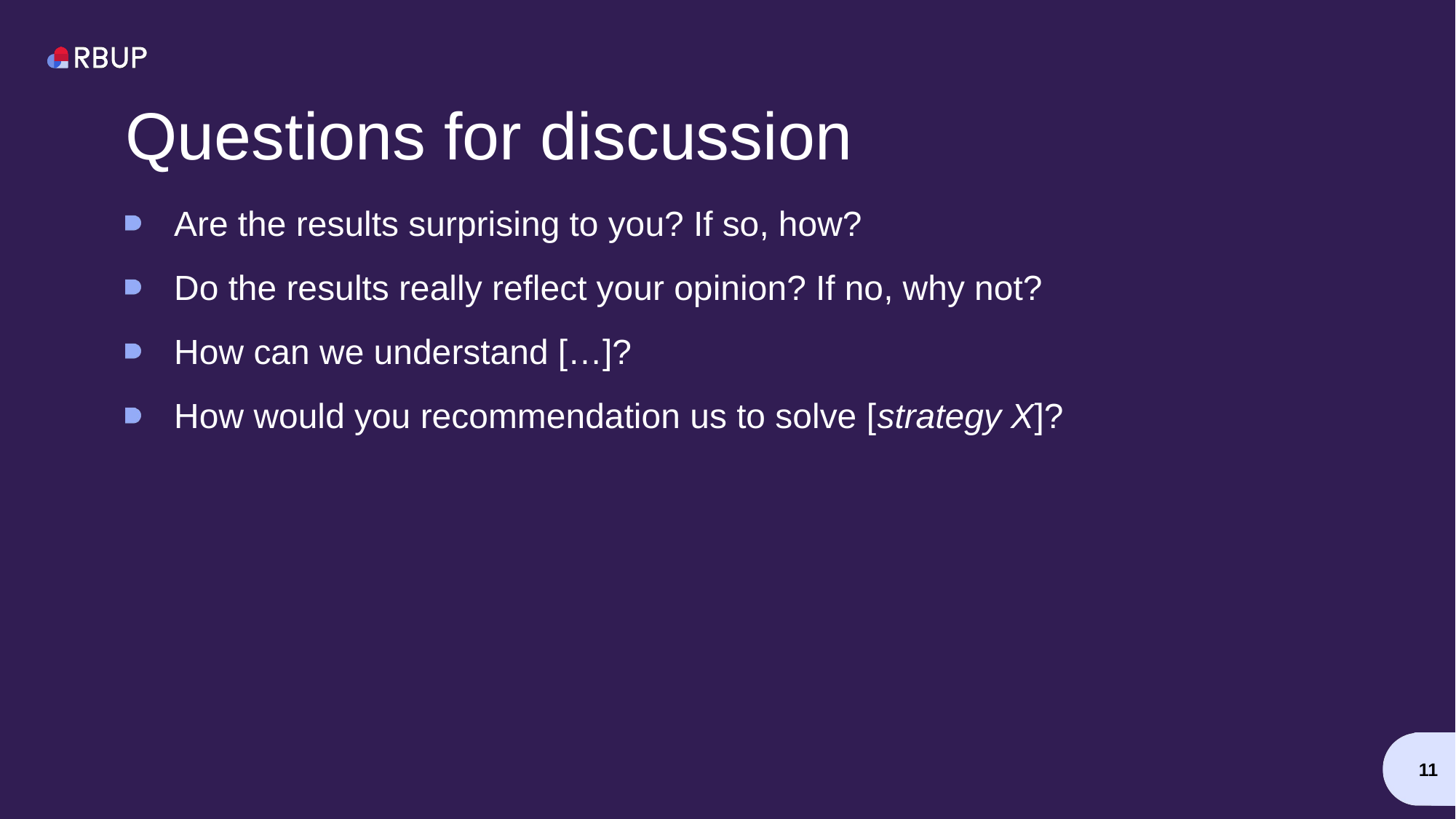

# Questions for discussion
Are the results surprising to you? If so, how?
Do the results really reflect your opinion? If no, why not?
How can we understand […]?
How would you recommendation us to solve [strategy X]?
11

## Slide 12
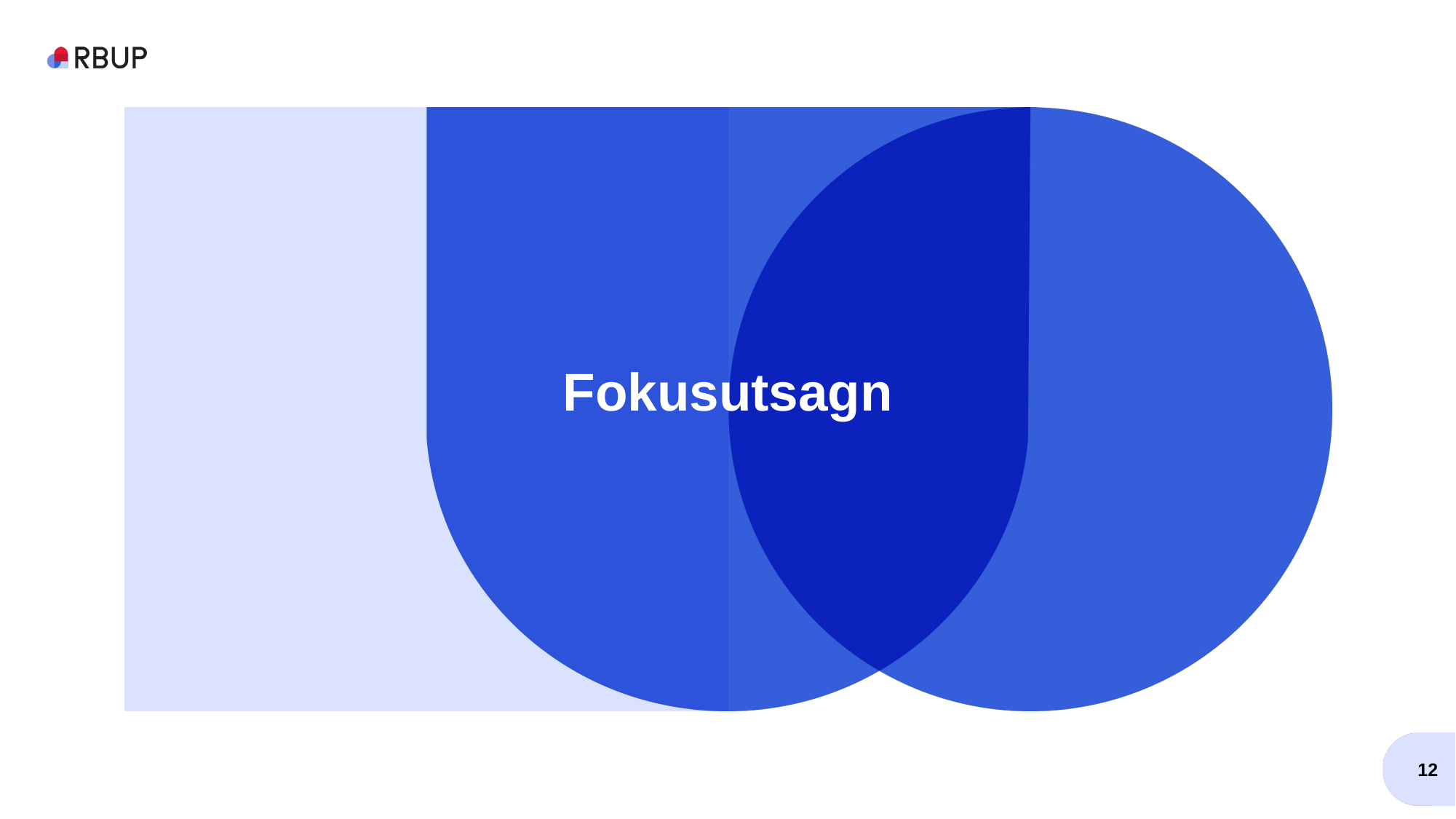

# Fokusutsagn
12

## Slide 13
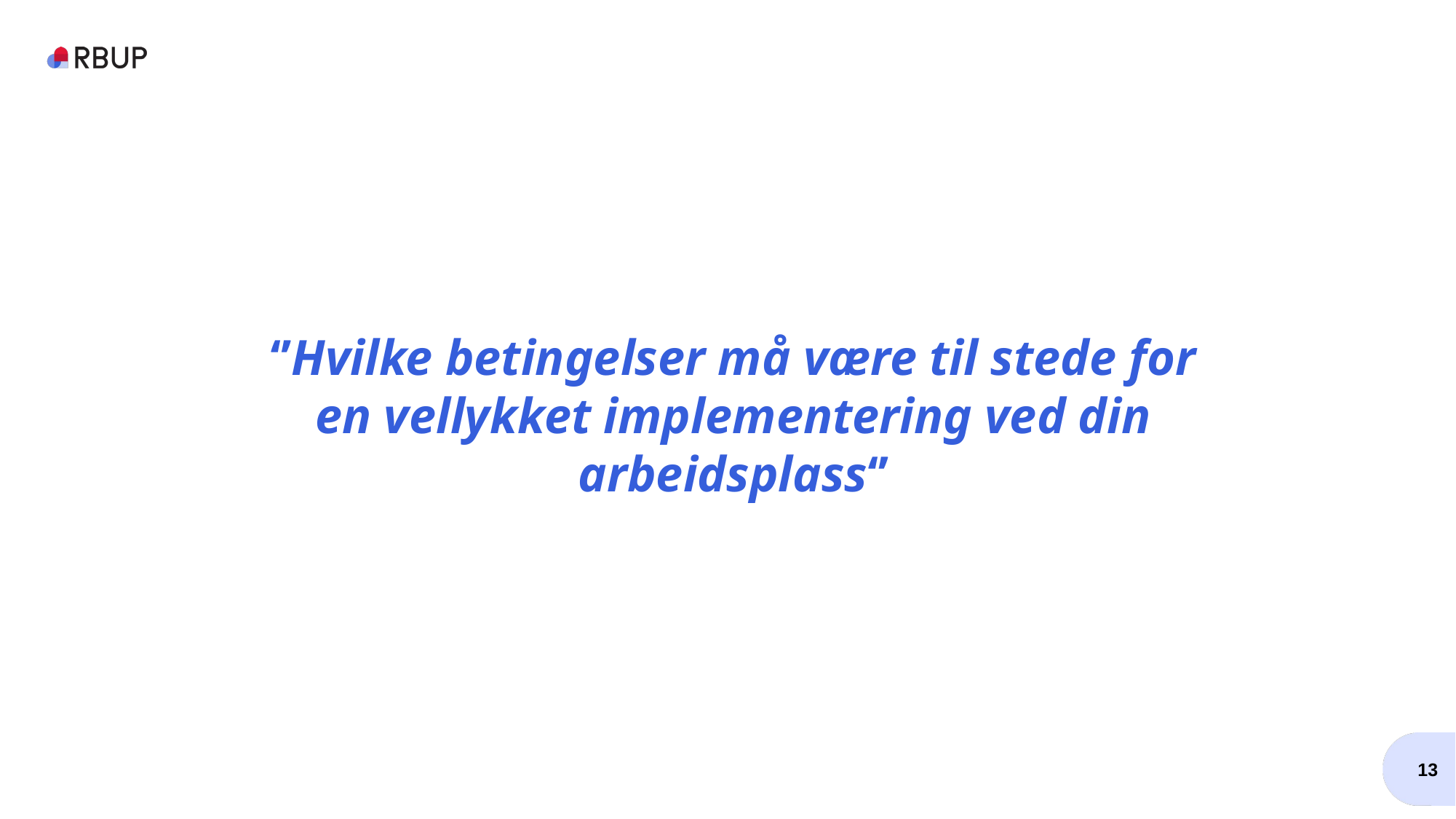

‘’Hvilke betingelser må være til stede for en vellykket implementering ved din arbeidsplass‘’
13

## Slide 14
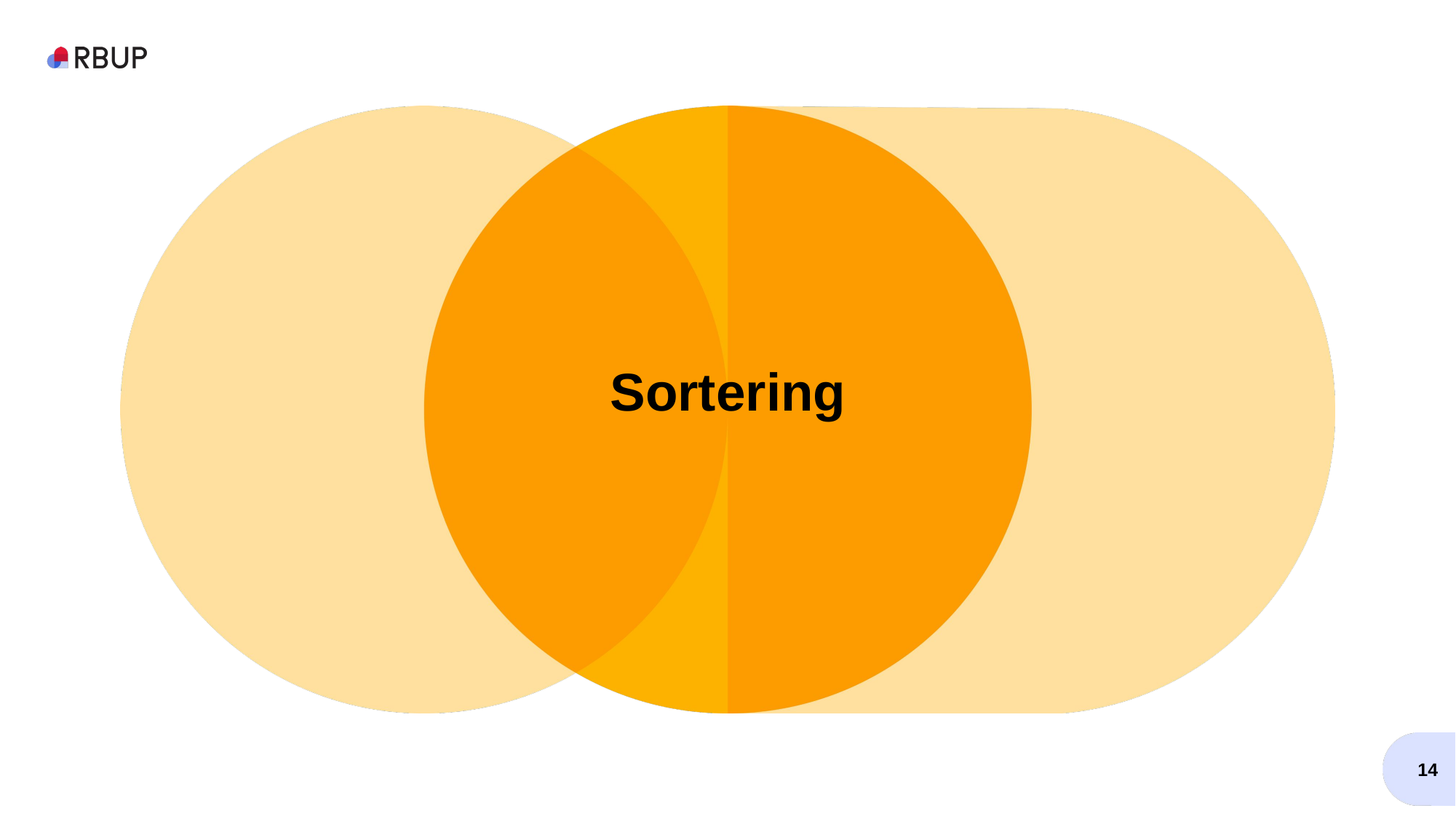

# Sortering
14

## Slide 15
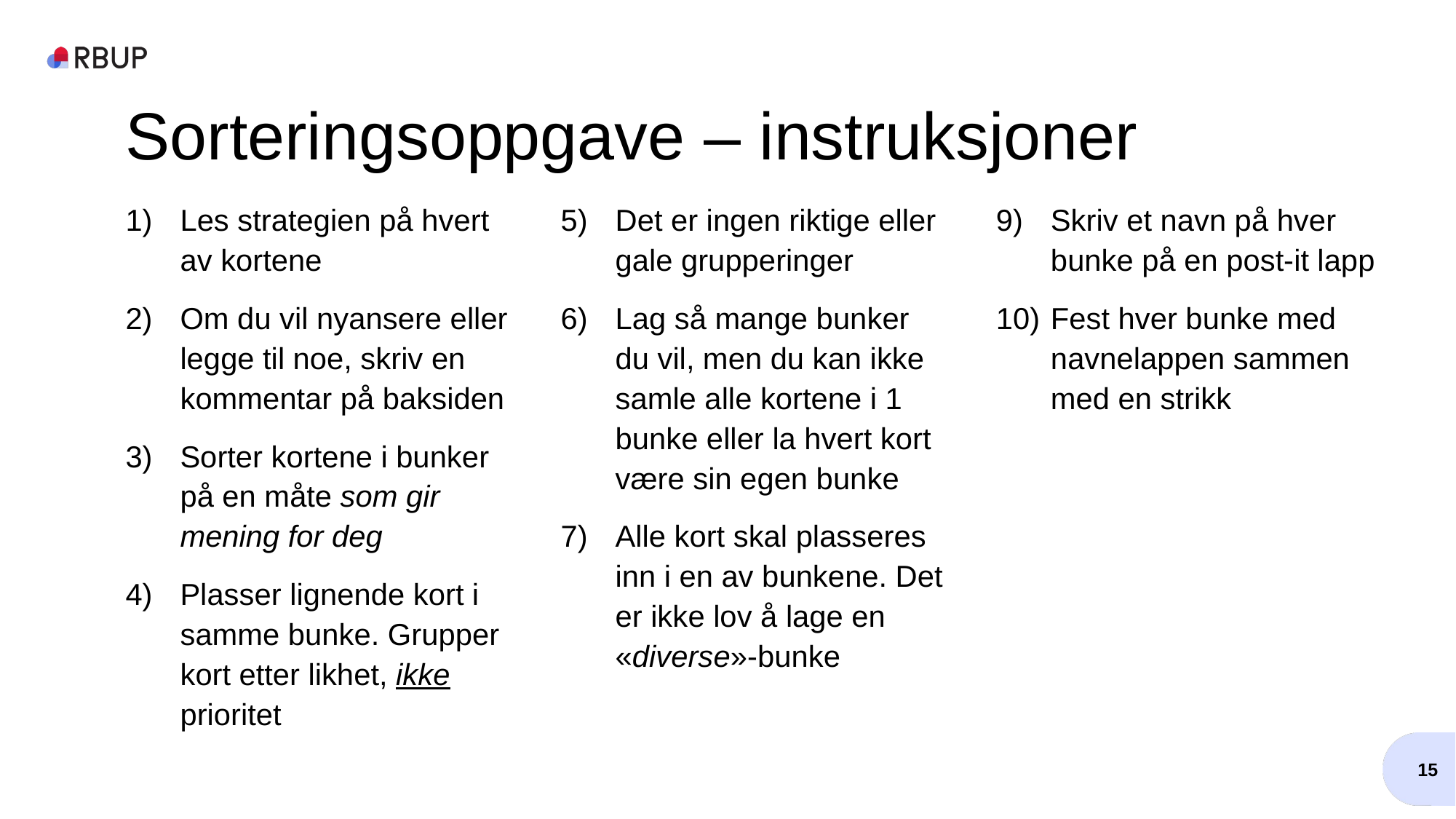

# Sorteringsoppgave – instruksjoner
Les strategien på hvert av kortene
Om du vil nyansere eller legge til noe, skriv en kommentar på baksiden
Sorter kortene i bunker på en måte som gir mening for deg
Plasser lignende kort i samme bunke. Grupper kort etter likhet, ikke prioritet
Det er ingen riktige eller gale grupperinger
Lag så mange bunker du vil, men du kan ikke samle alle kortene i 1 bunke eller la hvert kort være sin egen bunke
Alle kort skal plasseres inn i en av bunkene. Det er ikke lov å lage en «diverse»-bunke
Skriv et navn på hver bunke på en post-it lapp
Fest hver bunke med navnelappen sammen med en strikk
15

## Slide 16
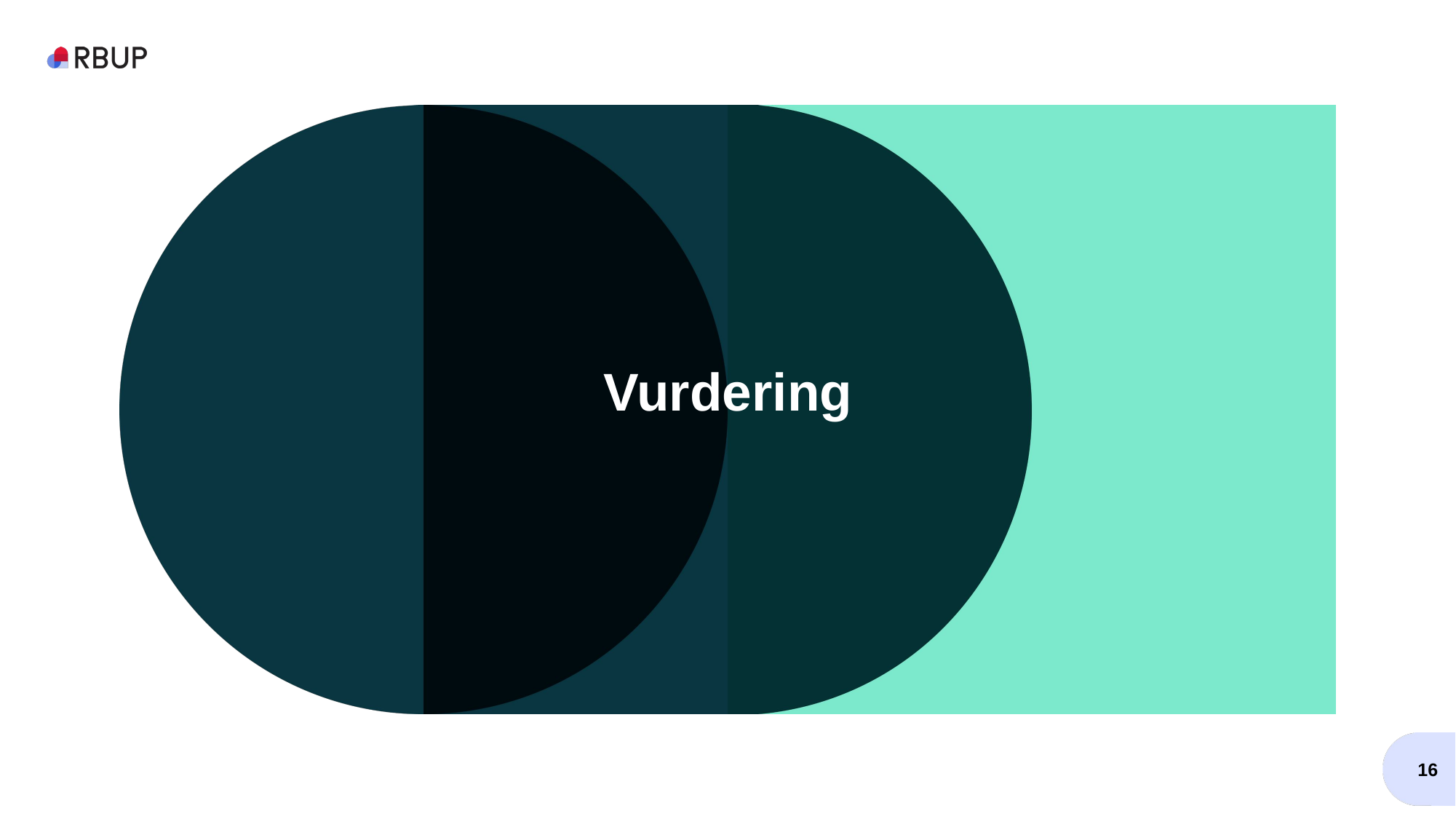

# Vurdering
16

## Slide 17
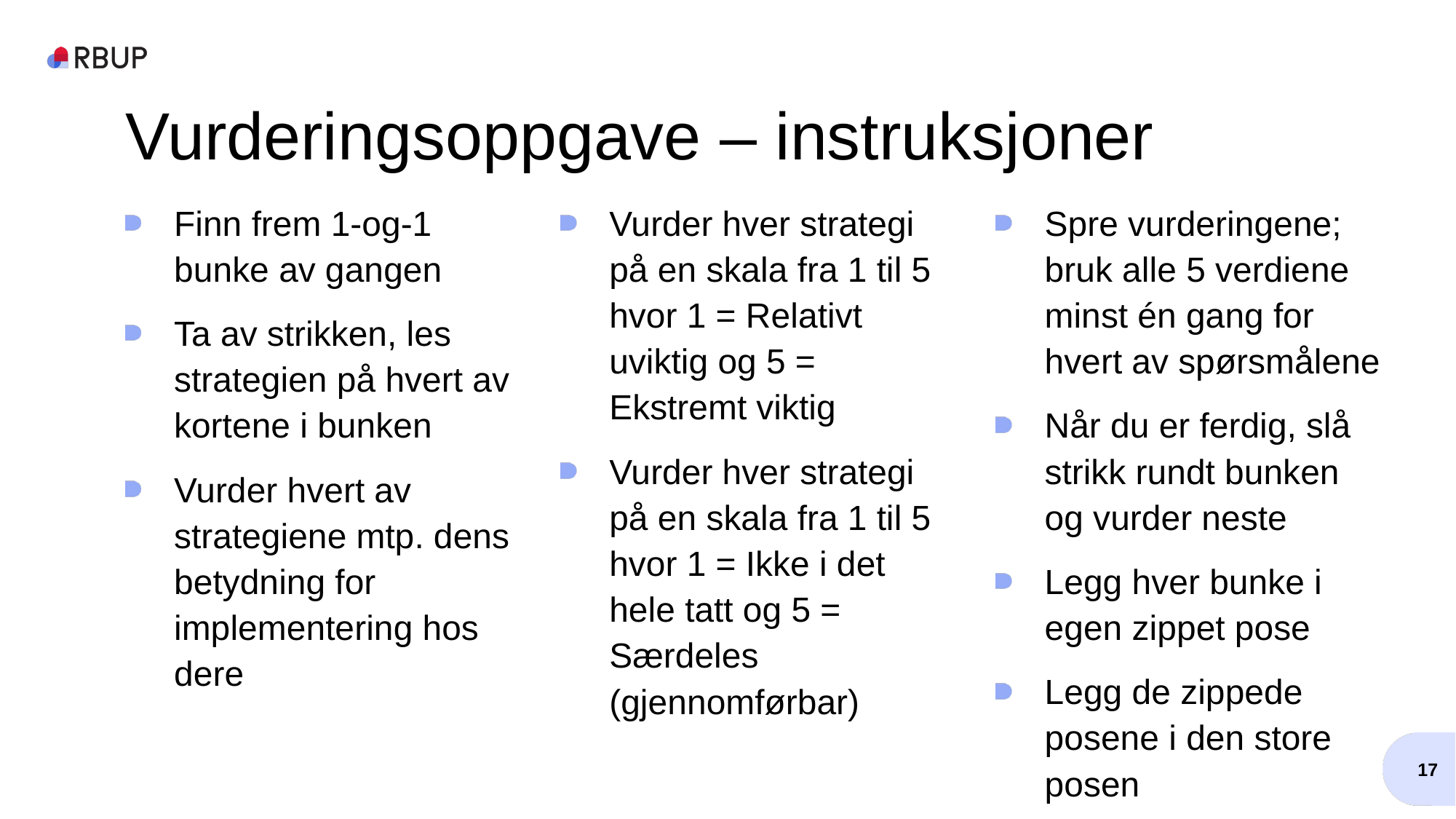

# Vurderingsoppgave – instruksjoner
Finn frem 1-og-1 bunke av gangen
Ta av strikken, les strategien på hvert av kortene i bunken
Vurder hvert av strategiene mtp. dens betydning for implementering hos dere
Vurder hver strategi på en skala fra 1 til 5 hvor 1 = Relativt uviktig og 5 = Ekstremt viktig
Vurder hver strategi på en skala fra 1 til 5 hvor 1 = Ikke i det hele tatt og 5 = Særdeles (gjennomførbar)
Spre vurderingene; bruk alle 5 verdiene minst én gang for hvert av spørsmålene
Når du er ferdig, slå strikk rundt bunken og vurder neste
Legg hver bunke i egen zippet pose
Legg de zippede posene i den store posen
17

## Slide 18
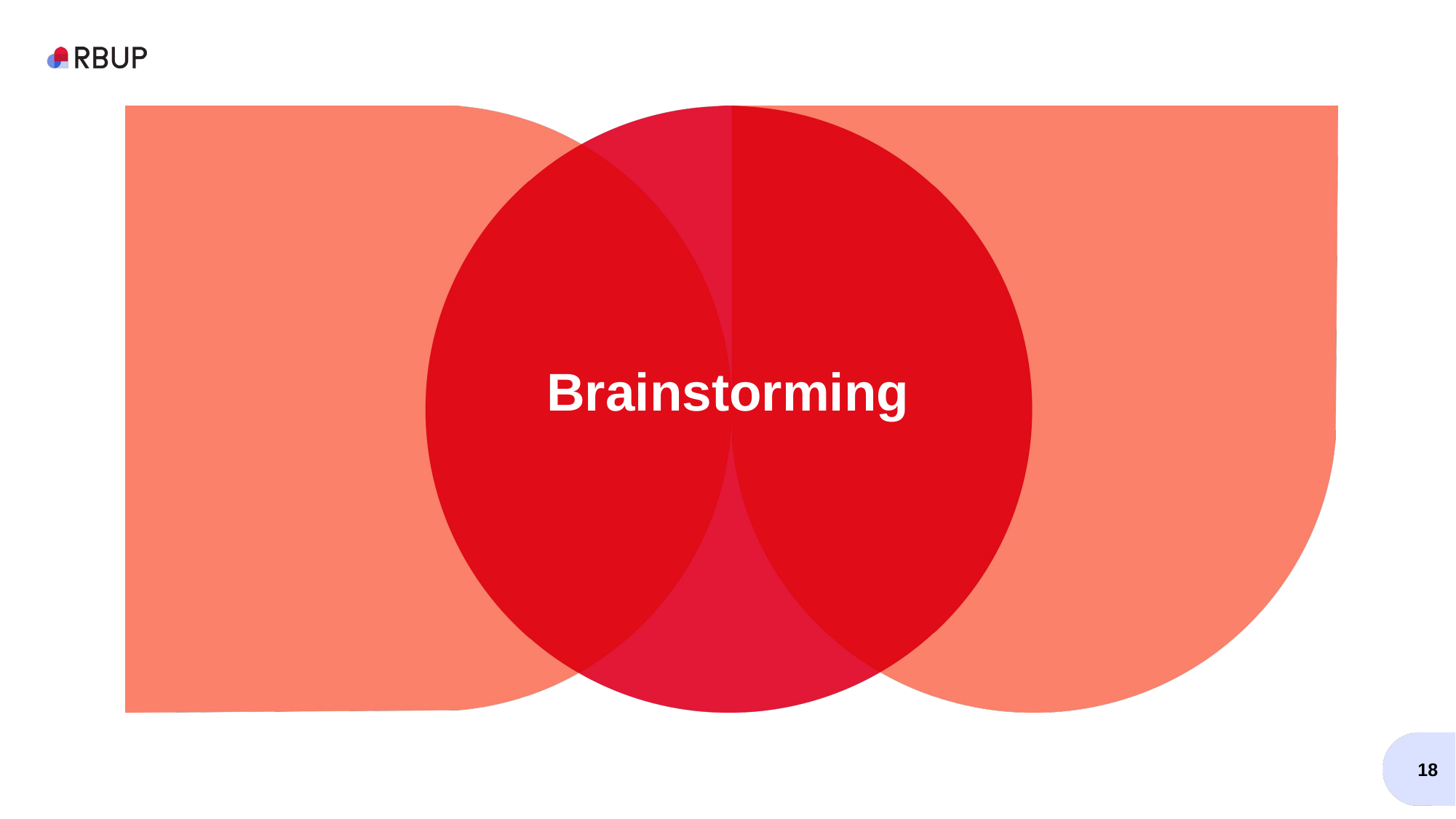

# Brainstorming
18

## Slide 19
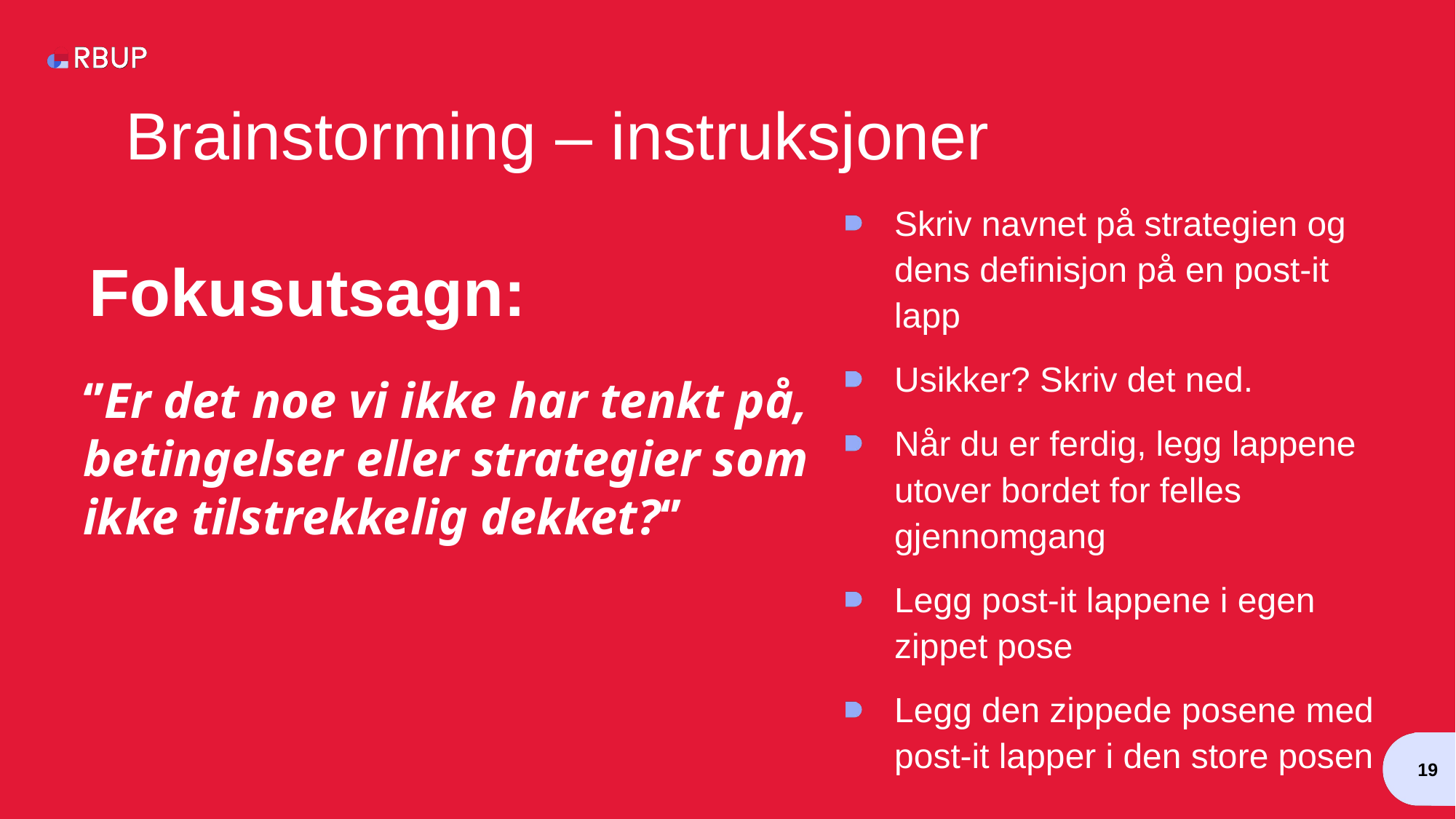

# Brainstorming – instruksjoner
Skriv navnet på strategien og dens definisjon på en post-it lapp
Usikker? Skriv det ned.
Når du er ferdig, legg lappene utover bordet for felles gjennomgang
Legg post-it lappene i egen zippet pose
Legg den zippede posene med post-it lapper i den store posen
Fokusutsagn:
‘’Er det noe vi ikke har tenkt på, betingelser eller strategier som ikke tilstrekkelig dekket?‘’
19

## Slide 20
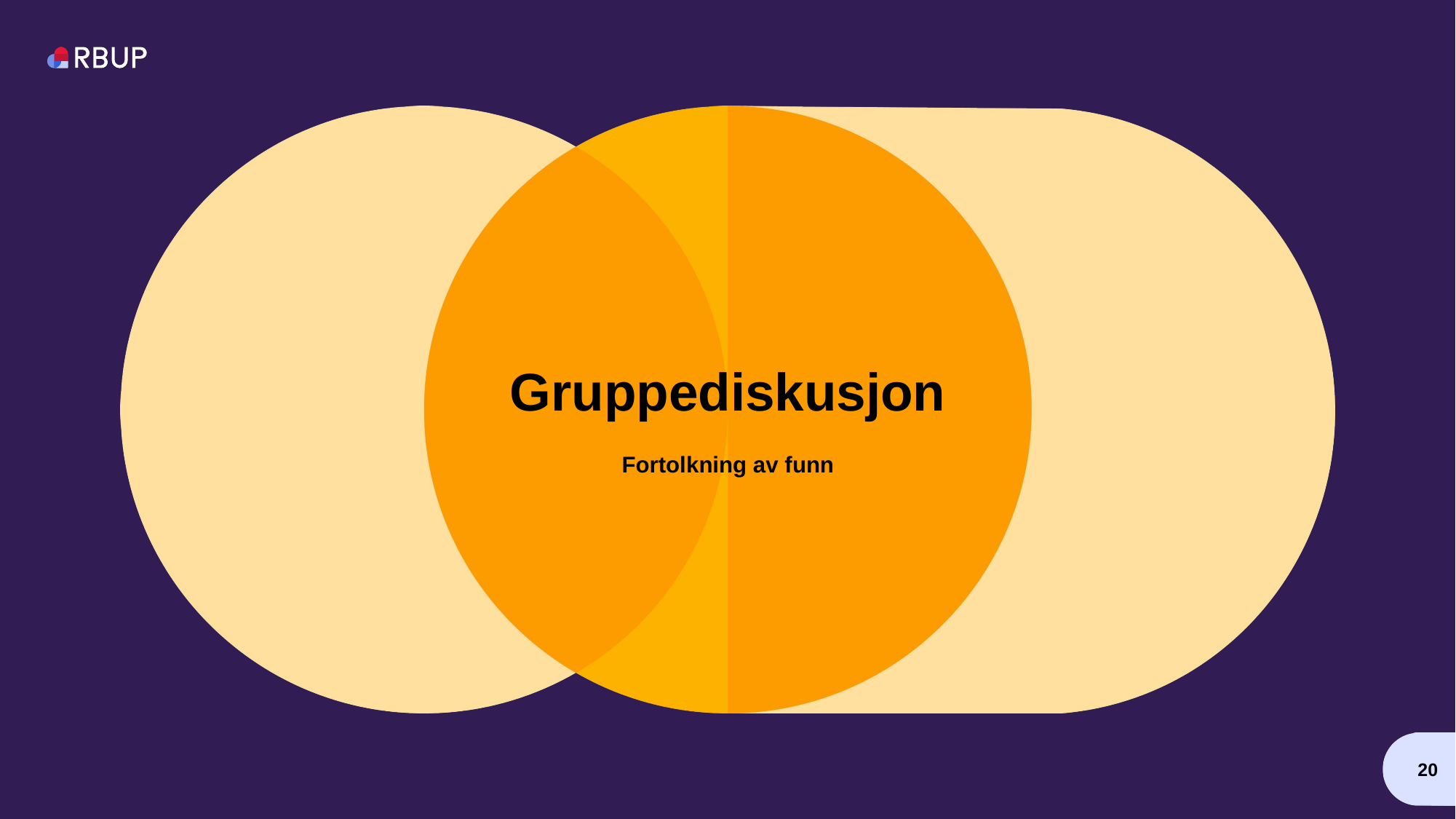

# Gruppediskusjon
Fortolkning av funn
20

## Slide 21
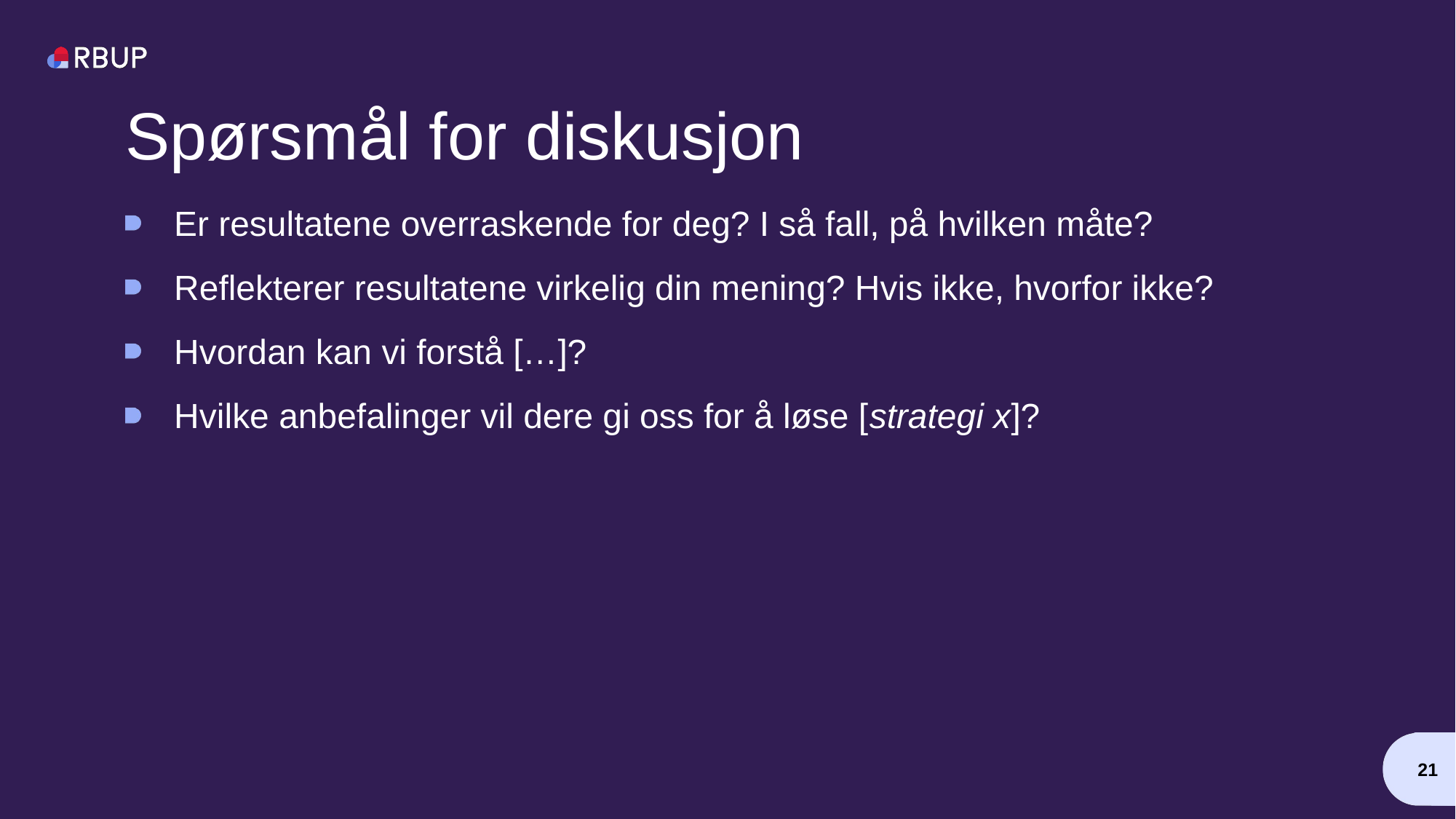

# Spørsmål for diskusjon
Er resultatene overraskende for deg? I så fall, på hvilken måte?
Reflekterer resultatene virkelig din mening? Hvis ikke, hvorfor ikke?
Hvordan kan vi forstå […]?
Hvilke anbefalinger vil dere gi oss for å løse [strategi x]?
21
